# An Investigation of Personal and Social-Contextual Resources as Mediators of the Influence of Duration of COVID-19 Pandemic Exposure and Mentoring Program Participation on the Mental Health and Well-Being of Adolescents

**DOI:** 10.3390/bs16101857

**Published:** 2026-10-09

**Authors:** David L. DuBois, Carla Herrera, Sara W. Kelly

**Affiliations:** 1 Institute for Health Research and Policy, University of Illinois Chicago, Chicago, IL 60607, USA; 2 Division of Community Health Sciences, University of Illinois Chicago, Chicago, IL 60607, USA; 3 Herrera Consulting, LLC, Washington, DC 20015, USA; carlaherrera66@gmail.com; 4 Department of Pediatrics, College of Medicine Peoria, University of Illinois Chicago, Chicago, IL 60607, USA; skelly88@uic.edu; 5 Institute for Research on Addictions, University of Illinois Chicago, Chicago, IL 60607, USA

**Keywords:** mentoring, COVID-19 pandemic, adolescents, mental health, well-being, personal resources, social-contextual resources

## Abstract

Although a sizable body of research indicates deterioration in the mental health of adolescents during the COVID-19 pandemic, the mediating processes accounting for these effects as well as the potential for community-based prevention programs to offset and/or buffer declines in mental health during this period have received limited investigation. The present study addressed these gaps using data from a randomized controlled trial of the Big Brothers Big Sisters of America Community-based Mentoring Program in which there was naturally occurring variation in the extent to which the 18-month window between baseline and an initial follow-up assessment overlapped with the pandemic. Structural equation modeling analyses revealed support for hypothesized indirect associations of pandemic exposure with negative change in mental health and well-being via co-occurring declines in levels of personal and social-contextual resources, as well as a partially offsetting indirect association of mentoring program participation with improved mental health via an increase in personal resources. Mentoring program participation did not interact with pandemic exposure, and model pathways generally did not vary significantly by gender. Findings suggest that declines in adolescent mental health during the COVID-19 pandemic were linked to disruptions in their personal and social-contextual resources and that community-based mentoring interventions may at least partially offset pandemic-related declines in mental health.

## 1. Introduction

The COVID-19 pandemic has been widely characterized as a substantial stressor for children and adolescents, with youth mental health subsequently designated a national emergency in the U.S. in 2021 (American Academy of Pediatrics et al., 2021). Importantly, however, evidence suggests that the deterioration in youth mental health did not originate during the pandemic itself. Rather, mental health indicators among U.S. youth were already worsening over the preceding decade (Blackwell et al., 2024). Between 2010 and 2023, the prevalence of depressive disorder more than doubled from 8.0% to 18.1% (Woolf, 2025). Parallel trends have been observed across other mental health indicators, including suicide mortality (Woolf, 2025). Taken together, these converging trends underscore a concerning pre-pandemic trajectory that likely reflects broader sociocultural and structural influences on youth well-being (Blackwell et al., 2024; Woolf, 2025).

Available evidence further suggests that these existing trends toward poorer mental health among youth may have accelerated during the early phases of the pandemic period, which was marked by uncertainty, social disruption, and rapid changes to daily life (Kauhanen et al., 2023; Samji et al., 2022; Theberath et al., 2022). A meta-analysis of 29 studies examining these patterns found that rates of anxiety and depression among children and adolescents nearly doubled compared to pre-pandemic estimates, suggesting an acute deterioration in mental health during the pandemic (Racine et al., 2021). This decline has been linked to several stressors. These include direct exposure to COVID-19 infection as well as indirect effects of the pandemic, including social isolation, family economic strain, and closure of schools and extracurricular activities, which together disrupted developmental routines and social interactions for youth (Zolopa et al., 2022).

As the pandemic evolved, public health surveillance and other reports indicate that youth mental health did not fully rebound to pre-pandemic levels, even as mitigation measures relaxed (Woolf, 2025; Baweja et al., 2026). In fact, recent studies suggest that elevated levels of depressive symptoms and reduced well-being, such as lower levels of positive affect and life satisfaction, have persisted into the post-pandemic period (Orban et al., 2024; Montero-Marin et al., 2023; Centers for Disease Control and Prevention, 2024). This pattern suggests a more sustained disruption in youth mental health, rather than a short-term response to acute stress exposure. Studies further suggest that these impacts were not uniform across populations. For example, mental health worsened disproportionately by gender (Samji et al., 2022), with the largest setbacks consistently documented in female youth, demonstrated by higher rates of depression, anxiety, and suicidality (Racine et al., 2021; Samji et al., 2022).

Against this backdrop, the present research seeks to further inform understanding of influences on mental health among youth in relation to the pandemic. More specifically, the research investigates the extent to which greater duration of pandemic exposure and participation in a mentoring program (the Big Brothers Big Sisters of America [BBBSA] Community-based Mentoring Program) were associated with potentially offsetting (i.e., negative and positive changes, respectively) in personal and social-contextual resources and the role of such changes in mediating associations between pandemic exposure and mentoring and co-occurring changes in indicators of positive mental health.

### 1.1. Personal and Social-Contextual Resources in the Context of the COVID-19 Pandemic

Both personal resources, such as social competence, adaptive coping, self-control, “grit” (i.e., adaptive persistence in the face of obstacles and pursuit of long-term goals), hope or positive expectations for the future, having a “spark” (i.e., a motivating special interest or hobby), and social-contextual resources, such as social support from peers, parents, and other adults, parenting practices (e.g., involvement and affirmation), and involvement in organized extracurricular activities, have, on the whole, a well-established role in supporting positive mental health (for recent reviews and primary studies, see Arseniou et al., 2026; Bashir & Peerzada, 2022; Baylon et al., 2024; Boelens et al., 2022; Gorostiaga et al., 2019, Kim et al., 2019; de Rooij et al., 2026; Rueger et al., 2016; Van Steenis et al., 2025). Furthermore, research suggests that such factors continue to be important for mental health during periods of large-scale societal disruption, including the COVID-19 pandemic. Studies conducted with adolescents during the pandemic, for instance, reported that higher levels of hope and positive expectations for the future were associated with lower levels of anxiety, depression and psychological distress (Rogers et al., 2024); greater reported hope and perceptions of being able to cope well with stress were associated with a lower likelihood of depression (Yu et al., 2026); and greater perceptions of self-efficacy, a factor with implications for perceptions of control, were predictive of greater life satisfaction in the wake of school closings (Göllner et al., 2026). Likewise, during the pandemic, greater levels of perceived social support were associated with fewer depressive symptoms (Liu et al., 2021), and family resilience (comprised of dimensions of harmony, openness, perseverance, and support) was associated with more favorable reported levels of mental health (Zhuo et al., 2022). Adolescents reporting higher levels of social connection during the COVID-19 pandemic similarly reported significantly fewer depressive symptoms and anxiety and significantly more life satisfaction (Magson et al., 2021), and those reporting higher levels of perceived general and parental social support were found to report greater life satisfaction over time, even during periods of significant pandemic-related restriction (Göllner et al., 2026). Collectively, these findings suggest that youth with higher levels of personal and social-contextual resources were less susceptible to declines in mental health and well-being during the pandemic.

Notwithstanding this evidence that personal and social-contextual resources fostered more positive mental health during the pandemic, it is equally important to consider how these resources themselves may have been disrupted during this time. Studies have documented declines in adolescents’ perceived social support and connectedness during pandemic-related school closures, reflecting reduced opportunities for interactions with peers, teachers, and other supportive individuals (Loades et al., 2020; Magson et al., 2021). Youth also reported deterioration in personal resources such as coping efficacy, optimism, and future orientation (Breaux et al., 2021; Samji et al., 2022). These findings suggest that adverse mental health outcomes of the pandemic may not be solely attributable to COVID-19-related stress exposure, but also to an erosion of personal and social resources that typically support healthy adjustment. More specifically, reductions in supportive relationships and opportunities for social engagement may represent key mechanisms linking pandemic-related disruption to subsequent declines in youth mental health. This possibility is consistent with developmental and resilience frameworks that emphasize that youth adjustment is shaped by exposure to adversity along with access to personal and environmental resources that facilitate successful adaptation (Masten, 2014; Fergus & Zimmerman, 2005). To date, we are not aware of research that has investigated pandemic-related declines in personal and social-contextual resources potentially mediating co-occurring changes in mental health among adolescents. However, consistent with this possibility, a longitudinal study of young adults in Korea (Noh & Park, 2022) found support for indirect effects of COVID-19-related stress on poorer mental health via negative changes in both resilience and social support.

### 1.2. Mentoring as a Promotive and/or Protective Intervention in the Context of the COVID-19 Pandemic

Along with advancing understanding of the implications that the COVID-19 pandemic may have had for personal and social-contextual resources of youth and their potential role in mediating co-occurring changes in youth mental health, it is equally important to examine the ways in which interventions provided to youth may function to offset and/or potentially alter these dynamics. One potentially beneficial intervention for youth exposed to the pandemic is the provision of social support through mentoring. Community-based mentoring programs aim to promote youth well-being through sustained, supportive relationships with non-parental adults. These programs pair youth with an adult to create a mentoring “match” that meets regularly in the community and engages in activities of their choosing (DuBois & Friend, 2017).

Pre-pandemic research provides evidence of positive effects of mentoring program involvement on mental health outcomes, including self-esteem, emotional symptoms, social anxiety, and depressive symptoms (DeWit et al., 2016; Herrera et al., 2023; King et al., 2021; Taussig et al., 2019). Research has also supported links between mentoring program involvement and benefits in areas relating to personal and social-contextual resources. These include improvements in personal resources such as positive future expectations and social competence as well as social contextual resources like experiences of social support and connectedness (see, e.g., DuBois et al., 2026; King et al., 2021; Karcher, 2008). There is also research pointing to mentoring-facilitated improvements in both personal and social-contextual resources being important in mediating improvements in youth mental health outcomes (Erdem et al., 2016; N. M. Hurd & Zimmerman, 2014; N. M. Hurd et al., 2014).

The extent and manner in which mentoring program participation may serve to promote personal and social-contextual resources and, thereby, confer mental health benefits in the context of an extremely stressful, life-changing event like the COVID-19 pandemic is a question that has only recently begun to be explored. One possibility is that participation in a mentoring program could benefit youth through promotive (offsetting) effects that accrue relatively independent of the degree of exposure to the stressor (in this case, pandemic exposure). Consistent with this possibility, studies have reported beneficial effects of mentoring even (and sometimes especially) for youth facing adversity. For example, studies have found favorable effects of mentoring program participation on self-determination, a personal resource, as well as mental health symptoms and quality of life for youth in foster care (Geenen et al., 2013; Taussig et al., 2019) and on social acceptance and depressive symptoms for youth experiencing high levels of adversity (i.e., “environmental risk”; Herrera et al., 2013). More broadly, meta-analyses have reported significant effects of mentoring programs among samples designated as “multi-risk” (Raposa et al., 2019) or high in environmental risk (DuBois et al., 2011).

A second possibility is that mentoring program participation could exert protective (buffering) effects by mitigating negative effects of stress exposure (again, in this context, the pandemic) on personal or social-contextual resources and mental health outcomes. Some evidence is consistent with this possibility. A study of 93 adolescent mothers, for example, found that the presence of mentors buffered the association between stress and mental health, with only those without a mentor showing the expected positive association between stress and depressive symptoms (N. Hurd & Zimmerman, 2010). Other research, however, has found little or no evidence of this type of stress-buffering effect for mentoring (see, for example, DuBois & Silverthorn, 2005; Sanchez et al., 2017). This pattern aligns with the broader social support literature for youth in which there has been limited evidence of stress-buffering effects for mental health outcomes, such as depressive symptoms (Rueger et al., 2016). Nonetheless, there is some evidence that suggests a role for mentoring in buffering the effects of stress associated with the pandemic. Specifically, in a study of 1232 11- to 18-year-old Black mentoring program participants (Douglas et al., 2025), COVID-related stress was marginally associated with disengaged coping at low levels of mentor support but not at high levels of mentor support.

A further important consideration is the disruptive effect that the COVID-19 pandemic had on mentoring programs. A survey of mentoring programs during the pandemic outlined several major challenges experienced by programs during this time (MENTOR: National Mentoring Partnership, 2020). Almost two thirds (65%) of the programs they surveyed had fundraising events cancelled; almost a third (30%) had previous financial commitments rescinded; and 29% had to decrease their staffing. Programs also had to contend with restrictions in their ability to recruit program participants from the community (Wright et al., 2024). Many programs were able to continue program operations by shifting from in-person to online meetings, but this was a potentially major shift for mentors and youth that were accustomed to meeting in person; safety considerations (e.g., additional or different types of background checks) and limited access to technology for many families also made the shift to e-mentoring challenging for programs (MENTOR: National Mentoring Partnership, 2020).

There is also evidence that the pandemic may have influenced the depth and quality of the interactions that mentors were able to engage in with youth. Mentors themselves, for example, had to adjust to the realities of the pandemic, confronting fear, anxiety, and changes in their own lives (Stark et al., 2021), a reality which may have detracted from their capacity to provide consistent and strong support for their mentees. Studies also have documented challenges with mentor-youth communication during the pandemic–for example access to technology and privacy concerns with online communication (e.g., Jarjoura et al., 2023; Kaufman et al., 2022). Mentors that shifted to online meetings also had to adapt to this new platform and communication methods (Stark et al., 2021). Mentors in one study described having to attend to their mentee’s non-verbal cues in a new way, relearn how to bond with their mentee, and in many cases, had to contend with new distractions in their mentee’s home setting (Stark et al., 2021), all of which may have affected the types and levels of social support provided in the relationship.

### 1.3. Present Study

**Figure 1:**
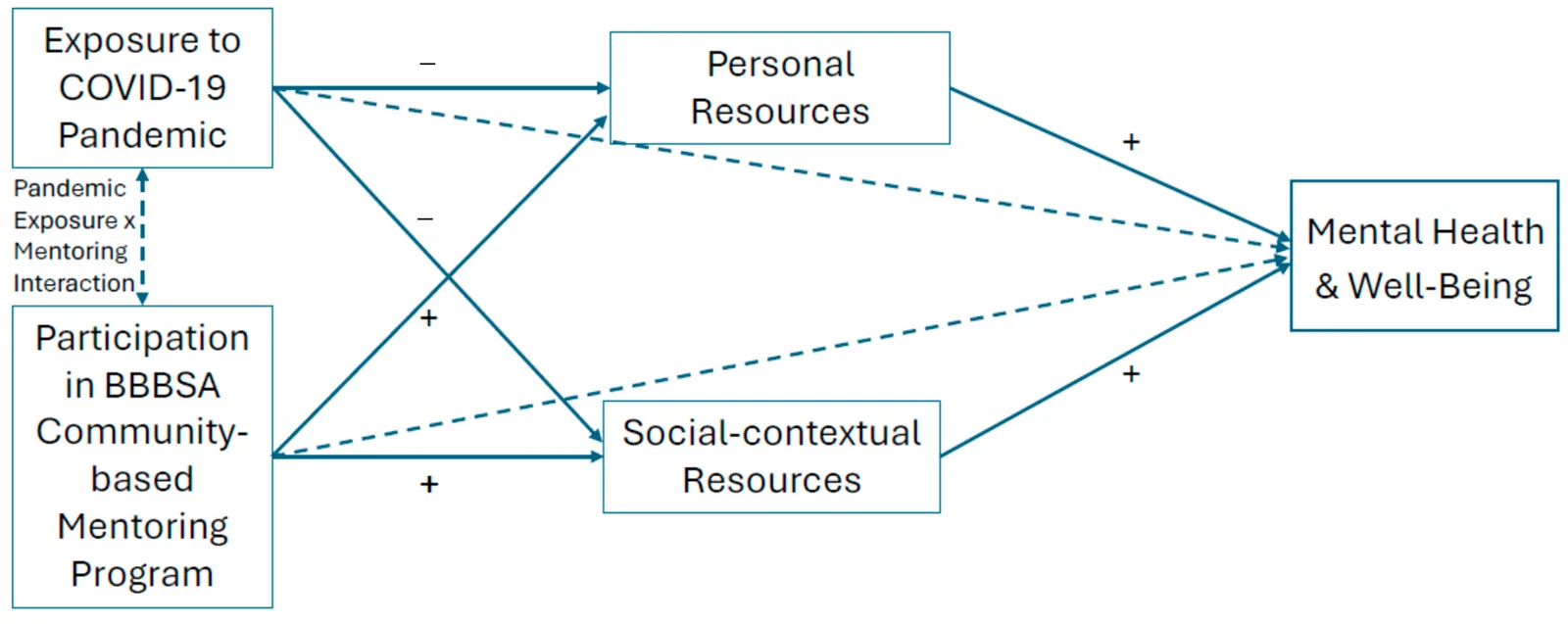
Conceptual model of effects of pandemic exposure and mentoring on youth mental health and well-being. Solid lines indicate hypothesized effects; dashed lines represent potential, but non-hypothesized, effects.

This study leverages data collected before and after the onset of the COVID-19 pandemic in a randomized controlled trial (RCT) of the BBBSA community-based mentoring program (the Youth Relationships Study; DuBois et al., 2026). In addition to study baseline, the trial included follow-up assessments of youth outcomes, via youth- and parent-report measures, at both 18 months and 4 years. By virtue of youth being enrolled in the study on a rolling basis from February 2018 to February 2020, about half had their 18-month assessment occur prior the start of the pandemic in March of 2020; the remainder had their 18-month follow-up occur at varying points in time after the onset of the pandemic. As a result, study data provides an opportunity not only to add to existing evidence of declines in mental health, as well as in personal and social-contextual resources, associated with exposure to the pandemic but also to examine effects of the BBBSA mentoring program on the same outcomes in relation to pandemic exposure (i.e., the two possibilities described above). The study also explores how youth experiences in the program (i.e., whether they were matched with a mentor and characteristics of the mentoring relationships that were established) may have been influenced by the pandemic. The primary aim of the study was to investigate the links depicted in the conceptual model shown in Figure 1. In line with the preceding review of research, the model posits that youth’s exposure to the pandemic (operationalized as how long the pandemic had been in effect at the time of their 18-month follow-up) has a negative effect on their mental health via more immediate disruptions to (i.e., reductions in) their personal and social-contextual resources. In a complementary manner, being randomly assigned to receive a mentor through the BBBSA mentoring program is hypothesized to improve the youth’s mental health via promotion of their social-contextual and personal resources. We thus expected estimates of indirect negative and positive effects of pandemic exposure and mentoring program participation on the mental health of youth to be statistically significant. The model thus reflected the expectation that benefits of mentoring program participation would at least partially offset the effects of pandemic exposure on mental health through opposing and independent pathways involving the same resources. The model allows for the possibility of direct effects of pandemic exposure and mentoring program participation on mental health as well as an interaction between pandemic exposure and mentoring program participation, the most likely pattern of which was expected to reflect stress-buffering in which negative effects of pandemic exposure on resources and mental health were mitigated by mentoring program participation.

In view of the evidence suggesting more severe effects of the pandemic on the mental health of girls, analyses also tested for possible moderation of model pathways by gender. Prior research has not revealed a strong and consistent pattern of differences in the impact of mentoring program participation for girls versus boys (Tierney et al., 1995). Some report patterns suggesting stronger benefits for boys (Park et al., 2017), whereas others report patterns suggesting stronger benefits for girls, particularly in school-based mentoring programs (Bernstein et al., 2009; Herrera et al., 2007). Nonetheless, gender differences in pathways involving mentoring program participation were explored alongside those involving pandemic exposure.

## 2. Materials and Methods

### 2.1. Sample

The sample for this research consists of the youth who participated in the above-described Youth Relationships Study. Study enrollment took place over a two-year period from February 2018 to February 2020. Study eligibility criteria consisted of: (a) youth being 10 years of age or older; (b) youth not having a sibling who was already a study participant, to avoid the potential for contamination if siblings were assigned to different groups; (c) youth not having a severe learning, cognitive, or other intellectual disability; (d) parent being able to read and speak either English or Spanish; (e) youth not having been previously matched with a mentor through any of the agency’s programs; (f) youth not having a sibling already receiving services from the agency; and (g) youth not falling into a group that the agency excluded from study participation based on agreement with the research team (e.g., some agencies chose not to recruit youth from one or more of their service regions). All youth also needed to be assessed by agency staff as meeting eligibility for participation in the program (e.g., living in the agency’s catchment area); agencies were, furthermore, permitted to exclude a small number of youth from study participation at their discretion to ensure immediate receipt of mentoring; 32 youth were excluded on this basis. Parent consent and youth assent for study participation were obtained for 1361 of the 1807 youth who met study eligibility criteria (and were not exempted by the agency), with the remainder who declined participation being eligible for matching with a mentor at the conclusion of the study enrollment period. An additional 3 youth withdrew from the study prior to being randomized and, as explained below, 5 youth were withdrawn following randomization, leaving a study sample of 1353 youth (see DuBois et al., 2026; for additional details on study recruitment and consent processes as well as the randomization and data collection procedures that are described in the following sections).

Demographically, the study participants were 62.7% male and, on average, were slightly older than 12 years of age (*M* = 12.30; *SD* = 1.54; interquartile range = 10.92 to 13.50, with age measured in fractional years based on date of birth). Youth had the following racial/ethnic backgrounds: Hispanic/Latine (30.2%), Black (38.7%), White (24.2%), and Other (6.9%). Nearly three-quarters (73.2%) were living in households with annual incomes that were below or within $5000 of the Federal poverty line.

### 2.2. Randomization

Youth participating in the Youth Relationships Study were randomized to study condition (i.e., treatment or control group) following completion of baseline study surveys. Youth were allocated to condition in a 3:1 ratio favoring the treatment group, with 1012 youth assigned to the treatment group (i.e., immediate eligibility to be matched with a mentor through the relevant BBBSA agency’s community-based mentoring program) and 346 assigned to the control group (i.e., eligibility for matching with a mentor delayed until the end of the 4-year study period). Three youth were withdrawn from the study following randomization by the researchers due to subsequently being determined to be ineligible due to age (*n* = 2) or cognitive ability (*n* = 1), and two parents withdrew their child in response to the child being randomly assigned to the control group. This resulted in a final study sample of 1353 youth, with 1011 in the treatment group (74.7%) and 342 (25.3%) in the control group. Youth assigned to the control group were able to participate in any activities that the agency provided for youth on the wait-list for mentoring; in addition, their parents were provided a list of referrals to non-mentoring youth programs in the community, a small stipend of $50 at the time of study enrollment, 18-month follow-up, and 4-year follow-up to support their child’s participation in these or other programs, and a tip sheet for connecting youth with supportive adults.

### 2.3. Data Collection

Following study enrollment, each youth’s parent completed a baseline survey independently while program staff administered a baseline survey to the youth (staff read questions aloud while the youth marked their responses on paper behind a privacy screen and then sealed their surveys in an envelope for forwarding to the researchers). The parent and youth each received a $10 incentive for completing these surveys. Families were then contacted by the research team 18 months and 4 years after the date of the youth’s study enrollment for the youth and parent to complete follow-up surveys. With few exceptions, youth and parents completed these latter surveys using REDCap, a secure web-based platform (P. A. Harris et al., 2009). At each follow-up, the youth and their parent each received $40 for completing their surveys. For the 18-month follow-up that is the focus of the present research, the survey response rates were 81.4% for youth and 84.8% for parents; both youth and parent response rates were comparable for treatment and control groups, with slightly higher rates for the control group (parent response rates of 84.1% and 87.0% and youth response rates of 80.6% and 83.5% for treatment and control groups, respectively). Neither parent nor youth response rates differed significantly between treatment and control groups, χ^2^(1) = 1.52, *p* = 0.216, and χ^2^(1) = 1.70, *p* = 0.192, respectively. Agencies provided information on if, and when, each youth in the treatment group was matched with a mentor during the study as well as the duration of these relationships.

### 2.4. Measures

Measures for the current study include assessments of personal resources, social-contextual resources, and mental health and well-being obtained at baseline and the 18-month follow-up (see Table S1 in the Supplementary Materials for details regarding each measure and sample reliability estimates). Measures of personal resources assessed self-control, conventional values, goal setting and pursuit, social skills, coping efficacy, spark development, grit, self-advocacy, and hopeful future expectations; all of these were assessed via youth-report, with the exception of self-control, which was derived from a combination of youth- and parent-report measures, and goal setting and pursuit, which was based on a parent-report measure. Measures of social-contextual resources assessed perceived social support from family members, friends, and a significant other (youth-report); family functioning, supportive parenting behavior, and the youth’s involvement in out-of-school activities (parent-report); and volunteering (youth-report). Measures of mental health and well-being assessed self-esteem, positive affect, and life satisfaction, all via youth self-report. Composite measures of changes in personal resources, social-contextual resources, and mental health between baseline and the 18-month follow-up were constructed. Each composite was created through a 3-step process. First, a series of regressions was carried out in which scores on each constituent measure (e.g., self-control in the case of personal resources) at the 18-month follow-up were regressed on the baseline scores for the measure as well as other variables that demonstrated evidence of a non-negligible (*p* < 0.10) difference between the treatment and control groups and/or association with the youth’s amount of pandemic exposure by the 18-month follow-up (see details regarding this measure below; see Table S2 in the Supplementary Materials for a list of baseline measures, inclusive of those from the larger Youth Relationships Study, that demonstrated evidence of association with BBBS mentoring and/or pandemic exposure and the relevant associations). Next, the saved residuals from these regressions, which captured change over time on the measure net of regression-to-the-mean tendencies and any change predicted by the above-referenced control measures, were standardized to a *z*-score format (i.e., mean of 0 and standard deviation of 1) to place them on a uniform scale. Finally, the resulting values for the relevant measures (e.g., self-esteem, life satisfaction, and positive affect in the case of mental health) were averaged to create the desired composite measure of change. An alternative approach would have been to use the residualized change scores for each set of measures as indicators of latent constructs (e.g., personal resources) in the structural equation modelling that was utilized to test the hypothesized conceptual model and associated research questions. However, this approach was deemed less desirable as it would have assumed that the scores on the various measures for each construct were emanating from a common latent construct. This assumption seemed less tenable than viewing the confluence of scores on the measures involved as reflecting a meaningful construct (e.g., rather than assuming there was a latent social-contextual resources construct that was a shared source of influence on qualitatively distinct experiences such as social support from friends and parenting behaviors, it was judged more plausible and appropriate to view the average of the scores involved as indicative of a relatively high or low overall level of social-contextual resources being available to the youth). From a statistical perspective, our approach assumed that the scores involved for each type of resource and mental health were more appropriately treated as forming the corresponding constructs rather than emanating from (i.e., reflecting) a common latent variable (for an in-depth discussion, see Bollen & Lennox, 1991).

The surveys completed by youth in the treatment group at the 18-month follow-up also included questions about the youth’s relationship with their mentor (“Big”) if the youth had been matched with one at any point prior to this time. If the youth’s mentoring relationship had ended prior to their completion of the 18-month survey, they were instructed to respond to the questions based on their experiences when the relationship was active. The questions and response options for each are provided in Table 1, as is the manner in which the responses were analyzed for reporting in preliminary analyses. Shortly after the onset of the pandemic, questions were added to the survey that parents of youth in the treatment group completed at the 18-month follow-up asking about modes of contact in the child’s mentoring relationship during the pandemic; additional questions asked about potential changes in the youth’s apparent feelings of closeness with their mentor and whether the mentor had provided the youth or parent with any assistance related to the pandemic. These questions, all of which were “yes” or “no” in response format, are shown in Table 2.

BBBS records were used to determine, as of the time of their 18-month follow-up, whether each youth in the treatment group had been matched with a BBBS mentor; the same records were used to determine if each match was still active at the time of the follow-up as well as its duration, regardless of whether it remained active. This information was used to compute the proportion of each match’s active period (up to the point of the youth’s 18-month follow-up) that occurred after the onset of the COVID-19 pandemic (i.e., 15 March 2020). For preliminary analyses (as described below), this information was used to distinguish youth with matches that were not active at all during the pandemic and those with proportions of post-pandemic active time of up to one third, between one and two thirds, or greater than two thirds. In view of the possibility that matches might be differentially affected as a function of whether they were already formed prior to the onset of the pandemic as well as whether they continued after this point in time, we also created a measure that distinguished between the following three groups of matched youth: (1) those with a match that both began and ended prior to the onset of the pandemic; (2) those with a match that began prior to the onset of the pandemic and was active after the pandemic’s onset; and (3) those with a match that began after the onset of the pandemic.

For both the preliminary and primary analyses (as described below), we compute the difference between the date of the youth’s 18-month follow-up (either the actual date of survey completion or, if the youth was a non-respondent, the scheduled date) and pandemic onset, expressed in fractional months. The resulting values indicate the amount of time between the youth’s study enrollment and 18-month follow-up that occurred after the start of the pandemic, with those with an 18-month follow-up that occurred prior to this time having scores of 0 on the variable (referred to hereafter as “pandemic exposure”).

### 2.5. Data Analyses

In preliminary analyses, we first examined potential associations between pandemic exposure and (a) whether youth in the treatment group had been matched with a mentor by the time of their 18-month follow-up and (b) for those who had been matched, the amount of time between study enrollment and matching as well as the overall length of the match. We then examined youth responses to the questions about their matches (coded as indicated in Table 1) and whether these varied significantly in relation to the above-described categorical variables reflecting the proportion of the match’s duration that occurred after the onset of the pandemic as well as the match’s time of initiation and duration of being active in relation to pandemic onset. In these latter analyses, we controlled for match duration, as analyses revealed a significant association between the measure of pandemic exposure and match length (see Section 3).

Pearson correlations were used to examine associations of the amount of pandemic exposure with both time to being matched and match length, as all the measures involved were continuous. Associations between pandemic exposure and the dichotomous variables of whether the youth was matched and whether the match remained active at the 18-month follow-up were assessed with point-biserial correlations. Logistic regression was used to examine each of the categorical variables that characterized matches in relation to pandemic onset as predictors of youth responses to the match questions, including match duration as an additional predictor to control for its influence. For ease of interpretation, parent responses to the dichotomous questions asked about matches during the pandemic were assessed in relation to the inverse of pandemic exposure—that is, the amount of time that had elapsed between the onset of the pandemic and when the parent was responding to the questions. These associations were assessed using point-biserial correlations.

Preliminary analyses also used independent group *t*-tests to examine potential gender differences in the composite measures of resources and mental health and well-being.

Primary analyses were carried out using version 9.1 of the Mplus software (Muthén & Muthén, 2026). These analyses began by fitting a structural equation model that corresponded to the conceptual model shown in Figure 1. Specifically, in this model, pandemic exposure and intervention group status (treatment = 1, control = 0; hereafter referred to as “BBBS mentoring”) were exogenous variables, allowed to correlate with each other, which each had paths to the composite measures of change in personal and social-contextual resources, allowing the error (disturbance) terms of the latter to correlate to reflect shared unexplained variance. The model also included paths from each resource measure to the composite measure of change in mental health and well-being. Finally, the model included direct paths from pandemic exposure and BBBS mentoring to the composite measure of change in mental health and well-being. These paths allowed for the possibility that changes in personal and social-contextual resources would not fully mediate effects of pandemic exposure and BBBS mentoring; the paths were also necessary from a technical perspective for appropriate estimation of the hypothesized indirect model pathways from pandemic exposure and BBBS mentoring to change in mental health and well-being (Schoemann et al., 2025). For the structural equation modeling analyses, measures of personal and social-contextual resources and mental health and well-being were standardized to a mean of 0 and standard deviation of 1 to facilitate interpretation of the magnitude of the estimated pathways.

To investigate the research questions that pertained to potential interactions of pandemic exposure and BBBS mentoring with each other and with gender, the structural equation model was expanded to include product terms between pandemic exposure and BBBS mentoring as well as between each of these variables and gender. Paths from the product terms to the measures of change in each type of resource and to change in mental health and well-being were then tested for significance.

In all analyses, *p* < 0.05 (two-tailed) was used as the threshold for statistical significance. Other findings with *p* values less than 0.10 were regarded as approaching significance. In analyses of the conceptual model, the indirect model pathways were evaluated using 95% confidence intervals that were constructed using the bias-corrected percentile bootstrap method with 1000 random samples. Missing data were handled using full-information maximum likelihood (FIML), as recommended by Schoemann et al. (2025), when testing mediation in the presence of missing data. Model fit indices were not applicable as the model was exactly identified.

Preliminary analysis revealed a trend approaching significance for those with 18-month follow-up data to have more pandemic exposure than those without follow-up data, *t*(1351) = 1.87, *p* = 0.061. The size of the difference was less than one month (*M*s of 5.26 months versus 4.57 months of exposure) and was in principle able to be accounted for by the inclusion of pandemic exposure as part of the FIML approach to handling missing data. However, as an additional safeguard, complete-case analyses (i.e., analyses limited to those with 18-month follow-up data) were conducted as a sensitivity analysis.

## 3. Results

### 3.1. Preliminary Analyses

Sample descriptive statistics for youth reports about their mentoring relationships are shown in Table 1. Youth in the treatment group with greater amounts of pandemic exposure were less likely to be matched by their 18-month follow-up (*r_pb_* [point-biserial correlation] = −0.094, *p* = 0.003). For illustrative purposes, 68.8% of those with no exposure (*n* = 282) had been matched compared to 67.3% of those with up to 6 months of exposure (*n* = 330) and 59.9% of those with more than 6 months of exposure (*n* = 397). Among those youth who had been matched, pandemic exposure was associated with the length of time between study enrollment and being matched with a mentor that approached significance (*r* = 0.075, *p* = 0.054); time to being matched was about one month shorter for those with no pandemic exposure (*M* = 3.29; *SD* = 3.39) relative to those with exposures of either up to 6 months (*M* = 4.22; *SD* = 4.39) or more than 6 months (*M* = 4.22; *SD* = 4.21). There was also a significant negative association with match duration (*r* = −0.147, *p* < 0.001), with longer duration for those with no pandemic exposure (*M* = 12.66; *SD* = 5.17) relative to those with up to 6 months of exposure (*M* = 12.14; *SD* = 5.35) and, to a greater extent, those with more than 6 months of exposure (*M* = 11.02; *SD* = 5.38). There was no association between pandemic exposure and whether matches were still active at the time of the 18-month assessment (*r_pb_* = 0.001, n.s.).

When examining potential variation in relationship characteristics based on the proportion of the match that came after the onset of the pandemic, there was a significant difference in the proportion of youth who reported at least every other week contact with their mentors (Wald chi-square (3) = 27.89, *p* < 0.001). Specifically, this level of contact was reported more frequently when either between one and two thirds (53%; *n* = 81) or more than two thirds (56%; *n* = 82) of the match’s active period was after the onset of the pandemic relative to when either none (29%; *n* = 257) or less than one third (31%; *n* = 86) of the match occurred post-pandemic. There was also an association with talking about social issues(Wald chi-square (3) = 8.63, *p* = 0.035), with this reported as occurring more frequently by those with matches with some, but less than one third of their match time occurring after the pandemic (44%) relative to the other three groups (range from 26% to 28%). All other associations between the proportion of match time after the pandemic and youth reports about their mentoring relationships did not reach or approach significance.

The three groups that were distinguished on the basis of the match’s time of initiation and time period of being active relative to pandemic onset differed in their reports of whether they had at least every other week contact with the mentor (Wald chi-square (2) = 9.29, *p* = 0.01). Those with matches that started after the onset of the pandemic were more likely to report this level of contact (65%; *n* = 34) than those with matches that started and ended prior to pandemic onset (37%; *n* = 136) or started before and ended sometime after pandemic onset (35%; *n* = 336). Differences between these groups did not reach or approach significance for any of the other measures.

Table 2 presents the percentages of parents reporting on different aspects of their child’s mentoring relationship when completing the 18-month follow-up during the pandemic, limiting consideration to those with children whose matches were active at this time (*n* = 190). Relative to those parents who responded further from the start of the pandemic, those who responded closer to its start were less likely to report their child was meeting with their mentor in person (*r_pb_* = −0.410, *p* < 0.001) and that the match was doing different types of activities together (i.e., not their traditional types of activities; *r_pb_* = −0.215, *p* = 0.003); in addition, there was a marginally significant trend for them to be more likely to report that their child was communicating with their mentor via phone (*r_pb_* = 0.132, *p* = 0.069) and that their child seemed to feel less close to their mentor (*r_pb_* = 0.141, *p* = 0.052).

Finally, when comparing scores on the composite measures on resources and mental health and well-being for males and females, females were found to have lower scores on the mental health and well-being composite, (*t*(1105) = −4.42, *p* < 0.001; females: *M* = −0.13, *SD* = 0.80; males: *M* = 0.08, *SD* = 0.79), whereas gender differences were not evident for either social-contextual resources (*t*(1182) = −0.51, n.s.) or personal resources (*t*(1172) = 0.14, n.s.).

### 3.2. Primary Analyses

**Figure 2:**
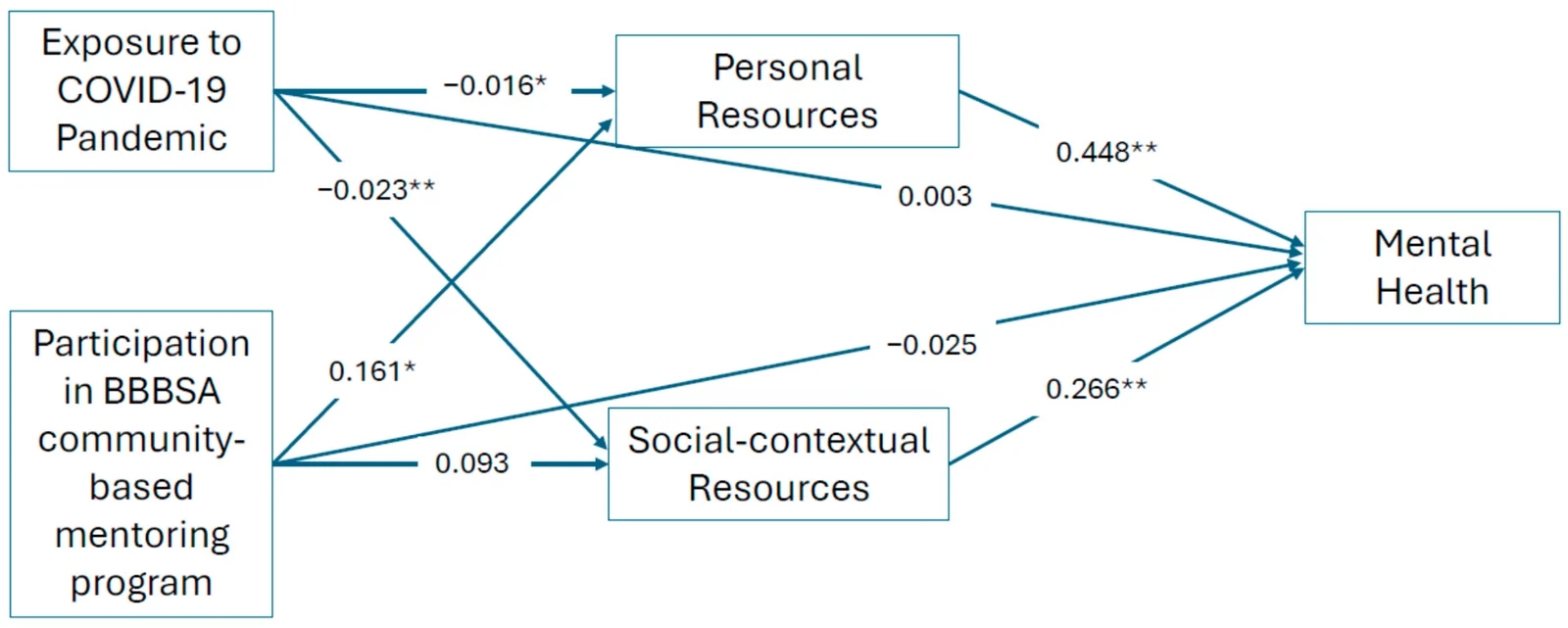
Unstandardized path estimates for the structural equation model testing the hypothesized conceptual model. Other model parameters are not shown but are provided in Table 3. * *p* < 0.05; ** *p* < 0.001.

As shown in Table 3 and Figure 2, the paths from pandemic exposure to changes in both personal and social-contextual resources were significant and in the hypothesized negative direction; the path from BBBS mentoring to change in personal resources was also significant and in the hypothesized positive direction, whereas the path from BBBS mentoring to change in social-contextual resources did not reach or approach significance. The paths from changes in each type of resource to change in mental health and well-being were significant and in the expected positive direction. Finally, the direct (unmediated) paths from pandemic exposure and BBBS mentoring to changes in mental health did not reach or approach significance.

Indirect pathway and total (direct + indirect) pathway estimates are shown in Table 4. Specific indirect pathways are those involving just one type of resource, whereas the total indirect pathway estimates are for both types of resources considered together. The specific indirect pathways linking pandemic exposure to negative change in mental health and well-being via concurrently assessed negative changes in personal and social-contextual resources were each significant, as indicated by the 95% confidence intervals for each pathway not including 0. The specific indirect pathway linking BBBS mentoring to improvement in mental health and well-being via change in personal resources was also significant. As expected, in view of the non-significant effect of BBBS mentoring on change in social-contextual resources, the specific indirect pathway from BBBS mentoring to mental health via this measure did not reach or approach significance. The total indirect pathway estimate for BBBS mentoring via both types of resources, however, was significant, as was the corresponding indirect pathway estimate for pandemic exposure. As can also be seen in Table 4, the overall (direct plus indirect) pathway estimates did not reach or approach significance. To summarize, linkages of pandemic exposure and BBBS mentoring to changes in mental health were evident only when the scope of estimates was limited to the hypothesized indirect linkages of BBBS mentoring and pandemic exposure to mental health via concurrent changes in resources.

As can be seen in Table 4, the estimated total indirect positive pathway from BBBS mentoring to mental health corresponded to a change over the 18-month study period of about one-tenth of a standard deviation (i.e., 0.097) and thus was small in magnitude. For six months of pandemic exposure, the estimated total indirect negative linkage between pandemic exposure and mental health approached the same magnitude (i.e., −0.098 based on 6 × the estimate of 0.013). Beyond this amount of pandemic exposure, the BBBS mentoring effect estimate was less than the estimated pandemic exposure–mental health linkage (e.g., for 12 months of exposure, it was 0.156) and thus was only partially offsetting.

The remaining primary analyses explored the potential interaction of BBBS mentoring and pandemic exposure with each other and with gender. With only one exception, none of the nine paths representing any of the three types of interaction (i.e., BBBS mentoring × pandemic exposure, BBBS mentoring × gender, pandemic exposure × gender) reached or approached significance. The exception was the interaction with gender for the direct path from BBBS mentoring to mental health (estimate = 0.222, *p* < 0.10). Allowing for this interaction resulted in the path being in a positive direction for females (estimate = 0.116, *p* = 0.178) and a negative direction for males (estimate = −0.013, *p* = 0.771), although neither path approached significance. Incorporating this interaction did not materially affect the estimates for the indirect paths from BBBS mentoring to mental health, with both the specific indirect path via personal resources and the total indirect path via both personal and social-contextual resources remaining significant for both males and females and the specific indirect path via social-contextual resources remaining non-significant for each gender. In this model, however, the total (direct + indirect) pathway for females was now significant (estimate = 0.213, 95% confidence interval from 0.020 to 0.410), owing to the stronger direct path from BBBS mentoring to mental health.

The results of the complete-case sensitivity analyses that were limited to youth with 18-month follow-up data closely mirrored those of the primary analyses, with the same path estimates being significant and of a very similar magnitude. Full results are available from the first author on request.

## 4. Discussion

Overall, the present findings are largely consistent with the hypothesized conceptual model in which mentoring provided through the BBBSA Community-based Mentoring Program and pandemic exposure were expected to have offsetting positive and negative influences on the mental health and well-being of study participants, in each case mediated by their more direct effects on personal and social-contextual resources. These results align with those of other recent randomized controlled trials that have indicated similar beneficial effects of both BBBS (Herrera et al., 2023) and other volunteer-based mentoring programs (e.g., Jarjoura et al., 2026; King et al., 2021) as well as a sizable body of research indicating that the COVID-19 pandemic took a measurable toll on the mental health of young people (Madigan et al., 2023a, 2023b; Racine et al., 2021). The current findings also extend these pieces of the literature in several notable ways. These include providing the most direct and rigorous evidence to date of a community-based prevention program being able to retain its effectiveness during the pandemic despite logistical and operational challenges and thus continue to function as an important source of support for participating youth during an especially challenging time in their lives. To our knowledge, the current study is also the first to provide a formal test of disruptions to personal and social resources as mediators of declines in mental health and well-being among youth during the pandemic. More generally, because the data were collected prior to the onset of the pandemic for some study participants and for others at varying points in the 18 months that followed, the research offers a novel contribution to existing evidence on the implications of the pandemic for developing youth. The COVID-19 pandemic, however, coincided with other societal developments that may have also influenced the mental health and resources of youth in the present sample. These include the widespread racial reckoning that was sparked by the murder of George Floyd and related events (Dyson, 2020). Thus, while our discussion of findings focuses on possible implications of variations in pandemic exposure, it should be kept in mind that the findings involved are correlational in nature and subject to potential confounding with co-occurring developments in the lives of the study’s youth.

These considerations having been noted, the findings of this study do contribute to a growing body of research suggesting that the effects of the pandemic on youth mental health may be at least partially attributable to the manner in which the pandemic disrupted personal and social-contextual resources in the lives of young people. This research has variously implicated school closures, social isolation, family stress, and interruptions to extracurricular activities in declines in youth mental health outcomes during the pandemic (Kauhanen et al., 2023; Orban et al., 2024; Wolf & Schmitz, 2024), as well as declines in specific areas of social-contextual and personal resources that align with those assessed in the present research. For example, fewer opportunities for interactions with peers and supportive adults outside the home, including disruptions in school and community activities, appear to have undermined these sources of support that have well-established importance for youth mental health (Loades et al., 2020; Magson et al., 2021; Martinsone et al., 2022). Other research has reported evidence of a decline in character (operationalized similarly to the conventional values measure used in the current study), potentially attributable to youth having fewer opportunities for socializing interactions with peers and teachers in the context of the pandemic (Kozina & Wiium, 2023) as well as to a decrease in positive future orientation (Rogers et al., 2024). Such findings suggest that mental health consequences of the pandemic were not solely a function of exposure to uncertainty and stressful circumstances but were also shaped by the ability to access personal and social-contextual resources needed to navigate those challenges. In fact, in the present study, once the links between pandemic exposure and reduced levels of each type of resource were taken into account, a direct pandemic exposure–mental health association was no longer evident.

Conversely, BBBS mentoring was linked to improved mental health through enhancement of personal resources, thus at least partially offsetting negative effects of the pandemic. In this context, it is useful to consider how the program and mentoring relationships may have been affected by the pandemic. Indeed, as would be expected, the pandemic was associated with a more limited capacity of agencies to match youth with mentors through the program. Specifically, those youth with greater exposure to the pandemic during the 18-month window between study enrollment and follow-up were less likely to have been matched with a mentor by their follow-up. This finding likely reflects agencies experiencing greater difficulty matching youth during the pandemic, with those youth who were enrolled closer to pandemic onset (and thus having greater pandemic exposure) being more susceptible to having been affected by these challenges (i.e., not having been matched by the time of pandemic onset). Consistent with this interpretation, the amount of time between program enrollment and being matched tended to be greater for those who had greater pandemic exposure and thus were enrolled closer to its start. This latter result likely accounts, in turn, for the finding that those with greater pandemic exposure by the 18-month follow-up had received fewer months of mentoring. The magnitude of these associations, however, was limited, and whether matches remained active at the youth’s 18-month follow-up was not related to whether the follow-up occurred after the onset of the pandemic. Parent reports also suggest that the quality of the relationships themselves was not markedly affected by the pandemic, with only a minority (22.7%) reporting that their child seemed less close to their mentor during the pandemic and a smaller proportion (15.5%) perceiving their child had become closer to their mentor. Of further note, pandemic exposure was associated with more frequent contact between mentors and their mentees. This finding is consistent with those of a qualitative study conducted in Italy (Marino et al., 2022) as well as Kaufman et al.’s (2022) study conducted early in the pandemic in which established matches were found to be continuing to meet as frequently as they had before the start of the pandemic.

Taken together, these findings suggest that despite considerable challenges, agencies were resilient and adaptive during this period in helping to ensure that youth had sustained opportunities for interaction with their mentors. Strategies such as facilitating online meetings and recommending activities suitable for that format were clearly an important part of these efforts (for related findings, see Wright et al., 2024). In view of the evidence that the pandemic was not a major source of disruption to BBBS mentoring, it is perhaps not surprising that findings indicate that participation in the program (i.e., assignment to the treatment group in the RCT) was of indirect benefit to the mental health and well-being of youth, as indexed by their reported levels of self-esteem, positive affect, and life satisfaction, via a relatively direct favorable effect on their personal resources, as indexed by the youth- and parent-report measures that formed the basis for this composite measure. Prior RCTs and quasi-experimental evaluations of mentoring programs for youth have reported effects on several of the same outcomes, such as social skills or competence (Resnjanskij et al., 2021) and hope or positive expectations for the future (Hellman & Gwinn, 2017; Kuperminc et al., 2011) in the case of personal resources, as well as self-esteem (Karcher, 2008; King et al., 2021; Marino et al., 2020) and life satisfaction (McQuillin & Lyons, 2016) in the case of mental health and well-being. Furthermore, in a white paper report of 18-month findings for the current trial, significant favorable effects were evident for five of the measures that constituted this study’s composite measure of personal resources (self-control, social skills, grit, self-advocacy, and hopeful future expectations). Yet, none of the effects on the individual measures comprising the composite measure of mental health and well-being (i.e., life satisfaction, self-esteem, and positive affect) reached or approached significance in these earlier analyses; in the present research, the overall (direct plus indirect) effect of mentoring program participation on the composite measure of mental health and well-being was similarly non-significant. This overall pattern of findings suggests that considering improvements in personal resources as a conduit to improvements in mental health and well-being was important for detecting benefits of mentoring program participation in this area. Such gains may provide a foundation for sustained improvements in mental health, potentially in part via reciprocal benefits for personal and social-contextual resources as suggested, for example, by research linking higher levels of self-esteem (M. A. Harris & Orth, 2020) and psychological well-being (Gómez-López et al., 2022) to improvements in social competence and relationships. In line with this possibility, the four-year follow-up assessment from the RCT that is the focus of the present research found significant effects on all three of the measures that contributed to the composite measure of mental health and well-being (DuBois et al., 2026).

Jarjoura et al.’s (2026) study of the YMCA’s Reach & Rise^®^ volunteer-based mentoring program overlapped with the pandemic period but did not find evidence of beneficial effects on measures of emotional well-being, including two of the constructs assessed in the current research (i.e., happiness and life satisfaction). It could be that examination of indirect effects on these outcomes via improvements in developmental assets or resources, as in the current research, would have revealed evidence of effects. The amount of mentoring received by the follow-up in the current study was also longer (close to a year vs. 8.8 months by Jarjoura et al., 2026). In addition, monthly check-ins with mentors are a standard of practice during the first year of the match in BBBS agencies (BBBSA, 2018), whereas only slightly more than half reported contacts with staff at this frequency in Jarjoura et al.’s (2026) study. These check-ins may have been particularly important during the pandemic (Marino et al., 2022).

We did not find evidence of effects of mentoring program participation on our composite measure of social-contextual resources. Similarly, in the earlier analyses that examined effects on the individual measures comprising this composite, a significant effect was found only for family functioning and for parental discipline, one of the four parenting measures that were averaged to form the composite. It could be that the environmental constraints associated with the pandemic limited the capacity of mentors to influence these types of resources, especially those that involved experiences outside of the youth’s home in the broader community (e.g., involvement in organized youth activities). This explanation is not fully supported, however, given the lack of an interaction between pandemic exposure and mentoring program participation. It also has not been uncommon to find non-significant effects on similar measures in other evaluations of mentoring programs. An earlier RCT of the same BBBS program that is the focus of the present research, for example, did not find a significant effect on a measure of parent–child relationship quality at the 13-month follow-up (Herrera et al., 2023), and Jarjoura et al. (2026) did not find evidence of an effect on a measure of peer relationship quality at the 15-month follow-up. It could be the case that a longer period of time is required for benefits to emerge in this area. Erdem et al. (2024), for example, found evidence in growth curve analyses that a difference in parent-reported family functioning favoring mentored relative to non-mentored youth in the BBBS program of Canada increased progressively across six time points that spanned a 2.5-year period. On the other hand, in the RCT that is the focus of this research, effects on the social-contextual measures generally continued to be non-significant at the four-year follow-up (DuBois et al., 2026).

We also found no evidence to indicate that the effects of mentoring program participation on outcomes were enhanced (or mitigated) as a function of pandemic exposure. Thus, although results are consistent with mentoring partially offsetting adverse effects of the pandemic on personal resources and mental health, it does not appear that it operated more directly on those latter effects to reduce or minimize them. In contrast, one previously noted cross-sectional study did suggest a potential role for mentoring in buffering the effects of COVID-19-related stress (Douglas et al., 2025). Yet, the present findings are in line with prior research in which elevated environmental risk or adversity was not found to moderate effects of volunteer-based mentoring program participation on outcomes, including measures of mental health (Herrera et al., 2013). Meta-analyses of youth mentoring program effectiveness have similarly either failed to find evidence of differential impact as a function of the risk levels of participating youth (Raposa et al., 2019) or have reported a pattern of relatively complex moderation involving level of individual risk or vulnerability factors (DuBois et al., 2011). A meta-analysis of the broader literature examining social support in relation to depression among youth (Rueger et al., 2016) similarly failed to find strong evidence of stress-buffering, whereas there was robust support for general benefits of social support.

A number of factors could have contributed to the lack of evidence for mentoring buffering effects of the pandemic (i.e., absence of a mentoring **×** pandemic interaction). It may be the case, for example, that the volunteer mentors in the BBBS program had limited ability to directly address pandemic-specific disruptions that contributed to worse mental health outcomes (e.g., reduced opportunity for spending time with peers). In line with this possibility, only a small minority of the parents who were surveyed during the pandemic reported that they were aware of their child’s mentor having provided support geared toward helping their child handle challenges related to the pandemic. Youth whose follow-up periods overlapped more with the pandemic were also less likely to have been matched with a mentor by follow-up, a factor that could have lessened the potential for buffering effects to occur among the treatment group. Finally, it is important to keep in mind that mentors were themselves undoubtedly experiencing pandemic-related stressors (Stark et al., 2021), a reality that for some may have lessened their capacity to directly support their mentees with navigating the challenges they were experiencing.

Consistent with prior research, we found that girls had significantly more negative change in mental health and well-being, as indexed by our composite measure, over the 18-month study period than did boys. Gender did not interact with pandemic exposure, however, in relation to mental health, either directly or indirectly via differential associations of pandemic exposure with change in personal or social-contextual resources. This aspect of our findings differs from prior research suggesting that the pandemic was more detrimental to the mental health of girls (e.g., Racine et al., 2021). Prior research, however, has been focused predominantly on negative aspects of mental health, such as symptoms of depression and anxiety, rather than positive aspects like those that were the focus of the current study. In a notable exception, a study of secondary school students in Hungary with a design similar to that of the present research (Major et al., 2024) found that self-esteem was stable over time for both boys and girls who were assessed twice over a one-year period that preceded the pandemic, whereas among a cohort that was assessed shortly before the lockdown and then approximately one year later, self-esteem actually increased for girls while remaining stable for boys. Another study of Japanese youth ages 6 to 17 (Goto et al., 2022) found that whereas girls reported lower self-esteem and emotional well-being than boys at relatively early points in the pandemic (May 2020 for both outcomes and September/October 2020 for self-esteem), there were no gender differences evident at later points in time up to the study endpoint of December 2021. A further noteworthy difference between the current sample and the predominantly general community samples of prior studies of mental health effects of the pandemic is that youth in the current study were predominantly from low-income backgrounds. It may be that, in the context of ongoing socioeconomic adversity, gender was relatively less influential in shaping youth responses to the pandemic.

Several limitations of the present study are noteworthy and merit attention in future research. First, although findings are largely consistent with the hypothesized role of changes in personal and social-contextual resources mediating effects of BBBS mentoring and pandemic exposure on changes in mental health and well-being, other explanations are possible. Assessing resources and mental health over multiple time points could help to better establish the hypothesized temporal ordering of changes in resources and mental health while also investigating potential reciprocal effects in the opposite direction. Second, although there was likely considerable variability in the extent and nature of pandemic-related stress that individual youth and their families were experiencing, data were not available to incorporate this information into study analyses. Doing so could better capture implications for youth outcomes. It could also help to clarify the capacity of mentoring relationships to buffer the effects of certain types or levels of pandemic-level stress. Third, regardless of enrollment timing relative to pandemic onset, at least one third of youth assigned to the treatment group had not been matched with a mentor by their 18-month follow-up. Similar rates of matching have been reported in prior research on BBBS and other similar mentoring programs for youth (see, e.g., DeWit et al., 2016; Jarjoura et al., 2026; Lindstrom Johnson et al., 2022). In the present study, losing contact with the youth’s family and difficulties recruiting volunteer mentors matched to the youth’s gender were the two most commonly reported factors leading to a youth not having been matched (DuBois et al., 2026). To maintain optimum rigor in estimating effects of BBBS mentoring, the present study used an intent-to-treat approach in which all youth assigned to the treatment group were included in analyses regardless of whether they had been matched with a mentor. A limitation of this approach, however, is that it may underestimate the benefits of mentoring received through the program. Future research should seek to address this through application of recommended methods, such as instrumental variable analyses, which were beyond the scope of the current research. Fourth, because resources and mental health were measured as formative composites for conceptual reasons, measurement error was not modeled, thus likely attenuating path estimates. Fifth, because the mental health composite comprised entirely youth self-report measures, as were several of the measures comprising the resource composites, some of the associations that were evident between resources and mental health may reflect shared reporter variance rather than substantive mediation. Sixth, although only one of the nine tests undertaken to explore possible interactions (i.e., mentoring × pandemic exposure, mentoring × gender, and pandemic exposure × gender in relation to each type of resource as well as mental health) reached or approached significance, these analyses were likely to have considerably less statistical power than those that evaluated the hypothesized direct paths between different measures in the model (McClelland & Judd, 1993). This consideration underscores the need for caution in interpreting the results for interactions and that they should not be regarded as strong evidence for a lack of mentoring × pandemic exposure buffering or gender differences in model pathways. Finally, as noted at the outset of the Discussion, it is important to interpret the present findings with awareness of other societal developments that unfolded in a similar time frame to the pandemic. These developments may have both interacted with pandemic-related experiences and exerted their own independent influence on youth in the study. The racial reckoning in the aftermath of the murder of George Floyd (Dyson, 2020) seems especially noteworthy in this regard, especially considering that a majority of the youth in the present study had racial and ethnic minority backgrounds. Greater attention to the role of these types of broader environmental forces in shaping youth development, along with those that are more proximal, such as mentoring, is a promising avenue for future research.

These limitations notwithstanding, the findings of this research remain significant as evidence of the benefits that community-based prevention programs can still have for youth during periods of social disruption even when those same programs may themselves be influenced and forced to adapt to the disruption that is taking place. It is noteworthy in this regard that the BBBSA Community-based Mentoring Program has been scaled successfully to serve a large number of youth from diverse backgrounds throughout the U.S. This level of reach is consistent with the potential for even relatively small, but measurable, benefits to mental health, such as those reported in the current research, to make a meaningful contribution to offsetting the type of negative population-level effects in this area that appear to have unfolded during the pandemic.

## Figures and Tables

**Table 1:** Questions asked of youth regarding their relationship with their mentor and sample descriptive statistics at the 18-month follow-up.

Question	Response Options	Study Measure	Sample % ^a^
How often have you and your Big usually gotten together in person?	We’ve never met like this Less than once a month Once a month Every other week Once a week More than once a week	Whether the youth reported getting together in person with their mentor at least every other week	39.9%
How often have you and your Big had contact with each other on the phone?	Never Less than every few months Once every few months About once a month About once a week More than once a week	Whether the youth reported this type of contact with their mentor at least once a month	33.6%
How often have you and your Big had contact with each other via email, text message, or social media (Facebook, Instagram, Snapchat, etc.)?	Never Less than every few months Once every few months About once a month About once a week More than once a week	Whether the youth reported this type of contact with their mentor at least once a month	36.8%
When you have spent time with your mentor, about how long did your outings or meetings usually last (this can include in-person, online and phone meetings)?	Less than an hour 1–2 h 3–4 h 5–6 h More than 6 h	Whether the youth reported having outings/meetings with their mentors of at least 3–4 h	46.9%
How close do you feel to your Big? Choose the one number that shows your feelings between “Not close at all” (1) and “Extremely close” (10).	Ten discrete response options with “Not at all close” for first option, and “Extremely close” for last option	Whether the youth provided a closeness rating of 8 or higher on a 1 to 10 scale	58.4%
The next questions ask about how often you and your Big have done different activities. My Big and I have…	Never Rarely Sometimes Often Very Often	Whether the youth reported engaging in each activity at least “sometimes”	
done sports or some other type of physical activity together (for example, rode bikes, went bowling, etc.)			42.6%
gone to a community event or activity (for example, visited a library or museum, attended a concert or art show, volunteered together, etc.)			33.6%
done something related to school (for example, worked on homework, gone to a school event together, talked about how things are going at school, etc.)			26.3%
hung out together (for example, watched TV, played video games, gone shopping, shared a meal, etc.)			56.4%
done a creative activity together (for example, cooking, drawing, crafts, etc.)			35.0%
participated in an activity with other Big Brothers Big Sisters program matches			19.8%
talked about my friends or family			57.8%
talked about things I like to do or am interested in			76.2%
talked about my future (for example, the kind of job I’d like to have, college, etc.)			54.1%
talked about social issues (for example, cur-rent events in the news, politics, etc.)			29.3%
talked about a personal concern or problem I’m experiencing			37.4%
talked about ways of being healthy (for ex-ample, exercising, making good eating choices, avoiding unsafe or risky behaviors, etc.)			45.4%

Note. *N* ranges from 521 to 529 due to varying numbers of non-respondents to each question and is limited to those youth in the treatment group who completed the 18-month follow-up survey and had been matched with a mentor by this point in time (even if the match was no longer active). ^a^ The percentages correspond to youth who reported the more frequent or intensive level of activity or, in the case of feelings of closeness, the higher level of closeness.

**Table 2:** Parent-reported aspects of their child’s relationship with their BBBS mentor during the pandemic.

Relationship Characteristic	% Endorsement
Not in contact	10.8%
Meeting in-person	33.5%
Getting together online (for example, Skype, Zoom, texting)	35.6%
Communicating via phone	49.0%
My child and his/her Big are doing different types of activities together (i.e., not their typical activities) because of the COVID-19 outbreak.	42.3%
My child seems to feel closer or more “connected” to his/her Big because of the COVID-19 outbreak.	15.5%
My child seems to feel less close or “connected” to his/her Big because of the COVID-19 outbreak.	22.7%
My child’s Big has said or done things to help my child with handling challenges related to the COVID-19 outbreak, such as feelings, schoolwork, etc.	17.0%
My child’s Big has said or done things to help me, as a parent, with handling challenges related to the COVID-19 outbreak.	7.2%

Note. Findings are based on responses of 194 parents of youth in the treatment group who completed the 18-month survey after the onset of the pandemic and whose children were in an active (i.e., not closed) match at that time (even if the youth and mentor were not currently in contact).

**Table 3:** Structural equation modeling estimates for the hypothesized conceptual model.

Parameter	Unstandardized Estimate	Standard Error	*z*	*p*
BBBS mentoring → personal resources	0.161	0.070	2.30	0.022
BBBS mentoring → social-contextual resources	0.093	0.065	1.42	0.156
Pandemic exposure → personal resources	−0.016	0.006	−2.58	0.010
Pandemic exposure → social-contextual resources	−0.023	0.006	−3.86	<0.001
Residual variance: personal resources	0.989	0.054	18.42	<0.001
Residual variance: social-contextual resources	0.984	0.043	22.81	<0.001
Covariance: residuals for personal and social-contextual resources	0.503	0.036	13.94	<0.001
Personal resources → mental health and well-being (MHWB)	0.448	0.038	11.74	<0.001
Social-contextual resources → MHWB	0.266	0.032	8.31	<0.001
BBBS mentoring → MHWB	−0.025	0.056	0.424	0.672
Pandemic exposure → MHWB	0.003	0.005	0.567	0.571
Residual variance: MHWB	0.653	0.029	22.53	<0.001

Note. *N* = 1353. Measures of personal and social-contextual resources and mental health and well-being were standardized to a mean of 0 and a standard deviation of 1 to facilitate interpretation of the magnitude of the estimates.

**Table 4:** Specific and total indirect and overall (direct plus indirect) pathways. Estimates and associated 95% confidence intervals.

Specific Indirect Pathway	Unstandardized Estimate	95% Confidence Interval
BBBS mentoring → personal resources → mental health and well-being (MHWB)	0.072	0.023, 0.127
BBBS mentoring → social-contextual resources → MHWB	0.025	−0.005, 0.056
Pandemic exposure → personal resources → MHWB	−0.007	−0.012, −0.002
Pandemic exposure → social-contextual resources → MHWB	−0.006	−0.003, −0.009
**Total Indirect Pathways**		
BBBS mentoring → MHWB via both personal resources and social-contextual resources	0.097	0.013, 0.175
Pandemic exposure → MHWB via both personal resources and social-contextual resources	−0.013	−0.005, −0.021
**Overall (Direct plus Indirect) Pathways**		
BBBS mentoring → MHWB	0.072	−0.071, 0.206
Pandemic exposure → MHWB	−0.011	−0.021, 0.002

Note. Measures of personal and social-contextual resources and mental health and well-being were standardized to a mean of 0 and standard deviation of 1 to facilitate interpretation of the magnitude of the estimated pathways. Additionally, 95% confidence intervals were constructed using the bias-corrected percentile bootstrap method with 1000 random samples.

## Data Availability

The data that support the findings of this study are available on request from the corresponding author with an executed data sharing agreement approved by each institution. The data are not publicly available due to privacy or ethical restrictions.

## Supplementary Materials

The following supporting information can be downloaded at: https://www.mdpi.com/article/10.3390/bs16101857/s1, Table S1: Measures of Personal Resources, Social-Contextual Resources, and Mental Health and Well-being; Table S2: Baseline Measures Exhibiting Associations (*p* < 0.10 or Stronger) with Intervention Group Status (BBBS Program or Control) and/or Pandemic Exposure. References (Arthur et al., 2002; Benson & Scales, 2009; Bowers et al., 2012; Cantril, 1965; Child Trends, 2022; DuBois et al., 1996, 2022; Duckworth & Quinn, 2009; Epstein et al., 1983; Essau et al., 2006; Forrest et al., 2018; Grasmick et al., 1993; Herrera et al., 2007, 2013; Jarjoura et al., 2023; Muris, 2001; Sandler et al., 2000; Zimet et al., 1988) are cited in the supplementary materials.

## Abbreviations

The following abbreviations are used in this manuscript:

BBBSA	Big Brothers Big Sisters of America
BBBS	Big Brothers Big Sisters

## References

[B1-behavsci-16-01857] American Academy of Pediatrics, American Academy of Child and Adolescent Psychiatry, Children’s Hospital Association (2021). Declaration of a national emergency in child and adolescent mental health.

[B2-behavsci-16-01857] Arseniou K., Gnardellis C., Lagiou A., Notara V. (2026). The role of family cohesion and health literacy in parents’ and adolescents’ health: A systematic review. Adolescents.

[B3-behavsci-16-01857] Arthur M., Hawkins J. D., Pollard J. A., Catalano R. F., Baglioni A. J. (2002). Measuring risk and protective factors for use, delinquency, and other adolescent problem behaviors: The Communities That Care Youth Survey. Evaluation Review.

[B4-behavsci-16-01857] Bashir N., Peerzada N. (2022). Social competence: A review analysis. Journal for ReAttach Therapy and Developmental Diversities.

[B5-behavsci-16-01857] Baweja R., Waschbusch D. A., Padilla F. M., Mattison R. E., Waxmonsky J. G. (2026). Post-pandemic trends in pediatric mental health treatment: A nationwide study. JAACAP Open.

[B6-behavsci-16-01857] Baylon N. K., Abrea L., Carpio M. A., Mesa N. E., Montesa J. A., Pusta C. E., Sugatan C. (2024). Grit and resilience as predictors of mental health outcomes of senior high school students. North American Journal of Psychology.

[B7-behavsci-16-01857] Benson P. L., Scales P. C. (2009). The definition and preliminary measurement of thriving in adolescence. The Journal of Positive Psychology.

[B8-behavsci-16-01857] Bernstein L., Rappaport C. D., Olsho L., Hunt D., Levin M. (2009). Impact evaluation of the US Department of Education’s student mentoring program *(Final Report, NCEE 2009-4047)*.

[B9-behavsci-16-01857] Big Brothers Big Sisters of America (2018). Big Brothers Big Sisters standards of practice for independent agencies.

[B10-behavsci-16-01857] Blackwell C. K., Wu G., Chandran A., Arizaga J., Bosquet Enlow M., Brennan P. A., Burton P., Bush N. R., Cella D., Cummins C., D’Sa V. A., Frazier J. A., Ganiban J. M., Gershon R., Koinis-Mitchell D., Leve L. D., Loftus C. T., Lukankina N., Margolis A., LeWinn K. Z. (2024). Longitudinal changes in youth mental health from before to during the COVID-19 pandemic. JAMA Network Open.

[B11-behavsci-16-01857] Boelens M., Smit M. S., Raat H., Bramer W. M., Jansen W. (2022). Impact of organized activities on mental health in children and adolescents: An umbrella review. Preventive Medicine Reports.

[B12-behavsci-16-01857] Bollen K. A., Lennox R. (1991). Conventional wisdom on measurement: A structural equation perspective. Psychological Bulletin.

[B13-behavsci-16-01857] Bowers E. P., Geldhof G. J., Schmid K. L., Napolitano C. M., Minor K., Lerner J. V. (2012). Relationships with important nonparental adults and positive youth development: An examination of youth self-regulatory strengths as mediators. Research in Human Development.

[B14-behavsci-16-01857] Breaux R., Dvorsky M. R., Marsh N. P., Green C. D., Cash A. R., Shroff D. M., Buchen N., Langberg J. M., Becker S. P. (2021). Prospective impact of COVID-19 on mental health functioning in adolescents with and without ADHD: Protective role of emotion regulation abilities. Journal of Child Psychology and Psychiatry.

[B15-behavsci-16-01857] Cantril H. (1965). The pattern of human concern.

[B16-behavsci-16-01857] Centers for Disease Control and Prevention (2024). Youth Risk Behavior Survey data summary & trends report: 2013–2023.

[B17-behavsci-16-01857] Child Trends (2022). Goal orientation scale.

[B18-behavsci-16-01857] de Rooij S. R., van Dijke-Dieleman L., Weinberg D., Vrijkotte T. G. M. (2026). Self-control during childhood and mental health at age 15–16: A longitudinal population-based study. European Child & Adolescent Psychiatry.

[B19-behavsci-16-01857] DeWit D. J., DuBois D., Erdem G., Larose S., Lipman E. L. (2016). The role of program-supported mentoring relationships in promoting youth mental health behavioral and developmental outcomes. Prevention Science.

[B20-behavsci-16-01857] Douglas R., Hsieh T. Y., Alvis L., Gaylord-Harden N., Syvertsen A. K. (2025). COVID-19 stress and coping among black youth: The role of socio-emotional community mentor support. Journal of Community Psychology.

[B21-behavsci-16-01857] DuBois D. L., Felner R. D., Brand S., Phillips R. S. C., Lease A. M. (1996). Early adolescent self-esteem: A developmental-ecological framework and assessment strategy. Journal of Research on Adolescence.

[B22-behavsci-16-01857] DuBois D. L., Friend D., Pepper K. (2017). Big Brothers Big Sisters. The SAGE encyclopedia of out-of-school learning.

[B23-behavsci-16-01857] DuBois D. L., Herrera C., Rivera J., Brechling V., Root S. (2022). Randomized controlled trial of the effects of the Big Brothers Big Sisters community-based mentoring program on crime and delinquency: Interim report of findings.

[B24-behavsci-16-01857] DuBois D. L., Herrera C., Rivera J., Brechling V., Root S. (2026). A randomized controlled trial of the Big Brothers Big Sisters community-based mentoring program. Annals of the New York Academy of Sciences.

[B25-behavsci-16-01857] DuBois D. L., Portillo N., Rhodes J. E., Silverthorn N., Valentine J. C. (2011). How effective are mentoring programs for youth? A systematic assessment of the evidence. Psychological Science in the Public Interest.

[B26-behavsci-16-01857] DuBois D. L., Silverthorn N. (2005). Natural mentoring relationships and adolescent health: Evidence from a national study. American Journal of Public Health.

[B27-behavsci-16-01857] Duckworth A. L., Quinn P. D. (2009). Development and validation of the Short Grit Scale (GRIT–S). Journal of Personality Assessment.

[B28-behavsci-16-01857] Dyson M. E. (2020). Long time coming: Reckoning with race in America.

[B29-behavsci-16-01857] Epstein N. B., Baldwin L. M., Bishop D. S. (1983). The McMaster family assessment device. Journal of Marital and Family Therapy.

[B30-behavsci-16-01857] Erdem G., DuBois D. L., Larose S., DeWit D. J., Lipman E. L. (2016). Mentoring relationships, positive development, and emotional and behavioral problems among youth: Investigation of a mediational model. Journal of Community Psychology.

[B31-behavsci-16-01857] Erdem G., DuBois D. L., Larose S., De Wit D. J., Lipman E. L. (2024). Associations of youth mentoring with parent emotional well-being and family functioning: Longitudinal findings from a study of Big Brothers Big Sisters of Canada. Children and Youth Services Review.

[B32-behavsci-16-01857] Essau C. A., Sasagawa S., Frick P. J. (2006). Psychometric properties of the Alabama Parenting Questionnaire. Journal of Child and Family Studies.

[B33-behavsci-16-01857] Fergus S., Zimmerman M. (2005). Adolescent resilience: A framework for understanding healthy development in the face of risk. Annual Review of Public Health.

[B34-behavsci-16-01857] Forrest C. B., Devine J., Bevans K. B., Becker B. D., Carle A. C., Teneralli R. E., Moon J., Tucker C. A., Ravens-Sieberer U. (2018). Development and psychometric evaluation of the PROMIS pediatric life satisfaction item banks, child-report, and parent-proxy editions. Quality of Life Research.

[B35-behavsci-16-01857] Geenen S., Powers L. E., Powers J., Cunningham M., McMahon L., Nelson M., Dalton L. D., Swank P., Fullerton A. (2013). Experimental study of a self-determination intervention for youth in foster care. Career Development and Transition for Exceptional Individuals.

[B36-behavsci-16-01857] Gorostiaga A., Aliri J., Balluerka N., Lameirinhas J. (2019). Parenting styles and internalizing symptoms in adolescence: A systematic literature review. International Journal of Environmental Research and Public Health.

[B37-behavsci-16-01857] Goto R., Piedvache A., Hangai M., Yamaoka Y., Sampei M., Sawada N., Okubo Y., Tanaka K., Morisaki N., Hosozawa M. (2022). Time trends in emotional well-being and self-esteem in children and adolescents during the COVID-19 pandemic. Child and Adolescent Psychiatry and Mental Health.

[B38-behavsci-16-01857] Göllner L. M., Rademacher J., Forstmeier S., Fassbender I. (2026). Adolescent wellbeing after COVID-related school-closings. Frontiers in Psychology.

[B39-behavsci-16-01857] Gómez-López M., Viejo C., Romera E. M., Ortega-Ruiz R. (2022). Psychological well-being and social competence during adolescence: Longitudinal association between the two phenomena. Child Indicators Research.

[B40-behavsci-16-01857] Grasmick H. G., Tittle C. R., Bursik R. J., Arneklev B. J. (1993). Testing the core empirical implications of Gottfredson and Hirschi’s general theory of crime. Journal of Research in Crime and Delinquency.

[B41-behavsci-16-01857] Harris M. A., Orth U. (2020). The link between self-esteem and social relationships: A meta-analysis of longitudinal studies. Journal of Personality and Social Psychology.

[B42-behavsci-16-01857] Harris P. A., Taylor R., Thielke R., Payne J., Gonzalez N., Conde J. G. (2009). Research electronic data capture (REDCap)—A metadata-driven methodology and workflow process for providing translational research informatics support. Journal of Biomedical Informatics.

[B43-behavsci-16-01857] Hellman C. M., Gwinn C. (2017). Camp HOPE as an intervention for children exposed to domestic violence: A program evaluation of hope, and strength of character. Child and Adolescent Social Work Journal.

[B44-behavsci-16-01857] Herrera C., DuBois D. L., Grossman J. B. (2013). The role of risk: Mentoring experiences and outcomes for youth with varying risk profiles *(A Public/Private Ventures Project)*.

[B45-behavsci-16-01857] Herrera C., DuBois D. L., Heubach J., Grossman J. B. (2023). Effects of the Big Brothers Big Sisters of America community-based mentoring program on social-emotional, behavioral, and academic outcomes of participating youth: A randomized controlled trial. Children and Youth Services Review.

[B46-behavsci-16-01857] Herrera C., Grossman J. B., Kauh T. J., Feldman A. F., McMaken J. (2007). Making a difference in schools: The Big Brothers Big Sisters school-based mentoring impact study.

[B47-behavsci-16-01857] Hurd N., Zimmerman M. (2010). Natural mentors, mental health, and risk behaviors: A longitudinal analysis of African American adolescents transitioning into adulthood. American Journal of Community Psychology.

[B48-behavsci-16-01857] Hurd N. M., Stoddard S. A., Bauermeister J. A., Zimmerman M. A. (2014). Natural mentors, mental health, and substance use: Exploring pathways via coping and purpose. American Journal of Orthopsychiatry.

[B49-behavsci-16-01857] Hurd N. M., Zimmerman M. A. (2014). An analysis of natural mentoring relationship profiles and associations with mentees’ mental health: Considering links via support from important others. American Journal of Community Psychology.

[B50-behavsci-16-01857] Jarjoura G. R., Herrera C., Tanyu M. (2023). Evaluation of reach & rise program enhancements to cognitive behavioral mentoring *(Technical Report)*.

[B51-behavsci-16-01857] Jarjoura G. R., Herrera C., Tanyu M., DuBois D. L. (2026). Effects of the Reach & Rise^®^ mentoring program on problem behavior and social-emotional and academic outcomes: A randomized controlled trial. American Journal of Community Psychology.

[B52-behavsci-16-01857] Karcher M. J. (2008). The study of mentoring in the learning environment (SMILE): A randomized evaluation of the effectiveness of school-based mentoring. Prevention Science.

[B53-behavsci-16-01857] Kaufman M. R., Wright K., Simon J., Edwards G., Thrul J., DuBois D. L. (2022). Mentoring in the time of COVID-19: An analysis of online focus groups with mentors to youth. American Journal of Community Psychology.

[B54-behavsci-16-01857] Kauhanen L., Wan Mohd Yunus W. M. A., Lempinen L., Peltonen K., Gyllenberg D., Mishina K., Gilbert S., Bastola K., Brown J. S. L., Sourander A. (2023). A systematic review of the mental health changes of children and young people before and during the COVID-19 pandemic. European Child & Adolescent Psychiatry.

[B55-behavsci-16-01857] Kim D. H., Bassett S. M., So S., Voisin D. R. (2019). Family stress and youth mental health problems: Self-efficacy and future orientation mediation. American Journal of Orthopsychiatry.

[B56-behavsci-16-01857] King C. A., Gipson P. Y., Arango A., Lernihan D., Clark M., Ewell Foster C., Caldwell C., Ghaziuddin N., Stone D. (2021). LET’s CONNECT community mentorship program for adolescents with peer social problems: A randomized intervention trial. American Journal of Community Psychology.

[B57-behavsci-16-01857] Kozina A., Wiium N. (2023). The development of the 5Cs of positive youth development in a school year: A 3-wave longitudinal study of Slovenian youth during the COVID-19 pandemic. Frontiers in Psychology.

[B58-behavsci-16-01857] Kuperminc G. P., Thomason J., DiMeo M., Broomfield-Massey K. (2011). Cool Girls, Inc.: Promoting the positive development of urban preadolescent and early adolescent girls. Journal of Primary Prevention.

[B59-behavsci-16-01857] Lindstrom Johnson S., Jones V., Ryan L. M., DuBois D. L., Fein J. A., Cheng T. L. (2022). Investigating effects of mentoring for youth with assault injuries: Results of a randomized controlled trial. Prevention Science.

[B60-behavsci-16-01857] Liu Y., Hu J., Liu J. (2021). Social support and depressive symptoms among adolescents during the COVID-19 pandemic: The mediating roles of loneliness and meaning in life. Frontiers in Public Health.

[B61-behavsci-16-01857] Loades M. E., Chatburn E., Higson-Sweeney N., Reynolds S., Shafran R., Brigden A., Linney C., McManus M. N., Borwick C., Crawley E. (2020). Rapid systematic review: The impact of social isolation and loneliness on the mental health of children and adolescents in the context of COVID-19. Journal of the American Academy of Child & Adolescent Psychiatry.

[B62-behavsci-16-01857] Madigan S., Korczak D. J., Vaillancourt T., Racine N., Hopkins W. G., Pador P., Jackson M. A., Hewitt, Batool A. M., McDonald S., Neville R. D. (2023a). Comparison of paediatric emergency department visits for attempted suicide, self-harm, and suicidal ideation before and during the COVID-19 pandemic: A systematic review and meta-analysis. The Lancet Psychiatry.

[B63-behavsci-16-01857] Madigan S., Racine N., Vaillancourt T., Korczak D. J., Hewitt J. M., Pador P., Park J. L., McArthur B. A., Holy C., Neville R. D. (2023b). Changes in depression and anxiety among children and adolescents from before to during the COVID-19 pandemic: A systematic review and meta-analysis. JAMA Pediatrics.

[B64-behavsci-16-01857] Magson N. R., Freeman J. Y. A., Rapee R. M., Richardson C. E., Oar E. L., Fardouly J. (2021). Risk and protective factors for prospective changes in adolescent mental health during the COVID-19 pandemic. Journal of Youth & Adolescence.

[B65-behavsci-16-01857] Major D., Falus M., Árva D., Eorsi D., Terebessy A., Tabak A. G., Fazekas-Pongor V. (2024). The effect of school lockdown on well-being and self-esteem of adolescents during the COVID-19 pandemic in Hungary. Frontiers in Public Health.

[B67-behavsci-16-01857] Marino C., Gaboardi M., Kaufman M. R., Bonichini S., Bergamin M., Canale N., Santinello M. (2022). “It is not necessary to organize a trip to the moon”: A mixed method study with mentors in the Italian Mentor-UP program during the COVID-19 pandemic. Mentoring & Tutoring: Partnership in Learning.

[B66-behavsci-16-01857] Marino C., Santinello M., Lenzi M., Santoro P., Bergamin M., Gaboardi M., Calcagnì A., Altoè G., Perkins D. (2020). Can mentoring promote self-esteem and school connectedness? An evaluation of the Mentor-UP project. Psychosocial Intervention.

[B68-behavsci-16-01857] Martinsone B., Stokenberga I., Damberga I., Supe I., Simões C., Lebre P., Canha L., Santos M., Santos A. C., Fonseca A. M., Santos D., Gaspar de Matos M., Conte E., Agliati A., Cavioni V., Gandellini S., Grazzani I., Ornaghi V., Camilleri L. (2022). Adolescent social emotional skills, resilience and behavioral problems during the COVID-19 pandemic: A longitudinal study in three European countries. Frontiers in Psychiatry.

[B69-behavsci-16-01857] Masten A. S. (2014). Resilience and positive youth development frameworks in developmental science. Journal of Youth & Adolescence.

[B70-behavsci-16-01857] McClelland G. H., Judd C. M. (1993). Statistical difficulties of detecting interactions and moderator effects. Psychological Bulletin.

[B71-behavsci-16-01857] McQuillin S. D., Lyons M. D. (2016). Brief instrumental school-based mentoring for middle school students: Theory and impact. Advances in School Mental Health Promotion.

[B72-behavsci-16-01857] MENTOR: National Mentoring Partnership (2020). Findings from MENTOR’s research into the impact of COVID-19 on mentoring programs and relationships.

[B73-behavsci-16-01857] Montero-Marin J., Hinze V., Mansfield K., Slaghekke Y., Blakemore S.-J., Byford S., Dalgleish T., Greenberg M. T., Viner R. M., Ukoumunne O. C., Ford T., Kuyken W. (2023). Young people’s mental health changes, risk, and resilience during the COVID-19 Pandemic. JAMA Network Open.

[B74-behavsci-16-01857] Muris P. (2001). A brief questionnaire for measuring self-efficacy in youths. Journal of Psychopathology and Behavioral Assessment.

[B75-behavsci-16-01857] Muthén L. K., Muthén B. O. (2026). *Mplus* (Version 9.1). Muthén & Muthén.

[B76-behavsci-16-01857] Noh D., Park S. (2022). Mediating effects of social support and resilience on the association between COVID-19-related stress and mental health in Korean young Adults. International Journal of Environmental Research and Public Health.

[B77-behavsci-16-01857] Orban E., Li L. Y., Gilbert M., Napp A.-K., Kaman A., Topf S., Boecker M., Devine J., Reiß F., Wendel F., Jung-Sievers C., Ernst V. S., Franze M., Möhler E., Breitinger E., Bender S., Ravens-Sieberer U. (2024). Mental health and quality of life in children and adolescents during the COVID-19 pandemic: A systematic review of longitudinal studies. Frontiers in Public Health.

[B78-behavsci-16-01857] Park H., Liao M., Crosby S. D. (2017). The impact of Big Brothers Big Sisters programs on youth development: An application of the model of homogeneity/diversity relationships. Children and Youth Services Review.

[B79-behavsci-16-01857] Racine N., McArthur B. A., Cooke J. E., Eirich R., Zhu J., Madigan S. (2021). Global prevalence of depressive and anxiety symptoms in children and adolescents during COVID-19: A meta-analysis. JAMA Pediatrics.

[B80-behavsci-16-01857] Raposa E. B., Rhodes J., Stams G. J. J. M., Card N., Burton S., Schwartz S., Yoviene Sykes L. A., Kanchewa S., Kupersmidt J., Hussain S. (2019). The effects of youth mentoring programs: A meta-analysis of outcome studies. Journal of Youth & Adolescence.

[B81-behavsci-16-01857] Resnjanskij S., Ruhose J., Wiederhold S., Woessmann L. (2021). Can mentoring alleviate family disadvantage in adolescence? A field experiment to improve labor-market prospects *(IZA discussion paper No. 14097)*.

[B82-behavsci-16-01857] Rogers E. M., Melde C., Williams J., Heinze J., McGarrell E. (2024). Adolescent mental health and resilience before and during the COVID-19 pandemic. Journal of Adolescent Health.

[B83-behavsci-16-01857] Rueger S. Y., Malecki C. K., Pyun Y., Aycock C., Coyle S. (2016). A meta-analytic review of the association between perceived social support and depression in childhood and adolescence. Psychological Bulletin.

[B84-behavsci-16-01857] Samji H., Wu J., Ladak A., Vossen C., Stewart E., Dove N., Long D., Snell G. (2022). Mental health impacts of the COVID-19 pandemic on children and youth – a systematic review. Child and Adolescent Mental Health.

[B85-behavsci-16-01857] Sanchez B., Mroczkowski A. L., Liao L. C., Cooper A. C., Rivera C., DuBois D. L. (2017). Mentoring as a mediator or moderator of the association between racial discrimination and coping efficacy in urban, low-income Latino/a youth. American Journal of Community Psychology.

[B86-behavsci-16-01857] Sandler I. N., Tein J. Y., Mehta P., Wolchik S., Ayers T. (2000). Coping efficacy and psychological problems of children of divorce. Child Development.

[B87-behavsci-16-01857] Schoemann A. M., Moore E. W. G., Yagiz G. (2025). How and why to follow best practices for testing mediation models with missing data. International Journal of Psychology.

[B88-behavsci-16-01857] Stark C., Wehrman J., Stutey D., Solis A., Severn K., Cammell M., Gloden H., Enkler K., Notestine, Palacios A. (2021). Therapeutic mentoring for multiply marginalized youth: A grounded theory study of mentors’ adaptation during the COVID-19 crisis. Journal of Human Services.

[B89-behavsci-16-01857] Taussig H. N., Weiler L. M., Garrido E. F., Rhodes T., Boat A., Fadell M. (2019). A positive youth development approach to improving mental health outcomes for maltreated children in foster care: Replication and extension of an RCT of the fostering healthy futures program. American Journal of Community Psychology.

[B90-behavsci-16-01857] Theberath M., Bauer D., Chen W., Salinas M., Mohabbat A. B., Yang J., Chon T. Y., Bauer B. A., Wahner-Roedler D. L. (2022). Effects of COVID-19 pandemic on mental health of children and adolescents: A systematic review of survey studies. SAGE Open Medicine.

[B91-behavsci-16-01857] Tierney J. P., Grossman J. B., Resch N. L. (1995). Making a difference: An impact study of Big Brothers/Big Sisters.

[B92-behavsci-16-01857] Van Steenis E., Boat A., Scales P. C., Arnold M. E., Ferrari T. M. (2025). Fostering youth sparks and social capital for greater equity in positive youth outcomes. Positive youth development: Integrating research and practice.

[B93-behavsci-16-01857] Wolf K., Schmitz J. (2024). Scoping review: Longitudinal effects of the COVID-19 pandemic on child and adolescent mental health. European Child & Adolescent Psychiatry.

[B94-behavsci-16-01857] Woolf S. H. (2025). The youth mental health crisis in the United States: Epidemiology, contributors, and potential solutions. Pediatrics.

[B95-behavsci-16-01857] Wright K., Levine D. K., Salcido M., Garringer M., Almendra T., Bazell A., Kaufman M. R. (2024). From crisis to capacity: Lessons learned from youth e-mentoring during the COVID-19 pandemic. Computers in Human Behavior Reports.

[B96-behavsci-16-01857] Yu Y., Chen J. H., Wu A. M. S., Zhang G., Xu D., Lyu X., Wang D. B., Du M., Lau J. T. F. (2026). Exploring why adolescents of disadvantaged family socio-economic status were more depressed than others: Serial mediations via personal psychological resources, loneliness, and school refusal functions. Journal of Affective Disorders.

[B97-behavsci-16-01857] Zhuo R., Yu Y., Shi X. (2022). Family resilience and adolescent mental health during COVID-19: A moderated mediation model. International Journal of Environmental Research and Public Health.

[B98-behavsci-16-01857] Zimet G. D., Dahlem N. W., Zimet S. G., Farley G. K. (1988). The Multidimensional Scale of Perceived Social Support. Journal of Personality Assessment.

[B99-behavsci-16-01857] Zolopa C., Burack J. A., O’Connor R. M., Corran C., Lai J., Bomfim E., DeGrace S., Dumont J., Larney S., Wendt D. C. (2022). Changes in youth mental health, psychological wellbeing, and substance use during the COVID-19 pandemic: A rapid review. Adolescent Research Review.

