# Supplementary material for: An Investigation of Personal and Social-Contextual Resources as Mediators of the Influence of Duration of COVID-19 Pandemic Exposure and Mentoring Program Participation on the Mental Health and Well-Being of Adolescents
Source: Behav Sci (Basel). 2026 Oct 9;16(10):1857. doi: 10.3390/bs16101857 (PMC13701299; doi:10.3390/bs16101857)
Supplement: Supplementary file 1 [file behavsci-16-01857-s001.zip › Table S1.pdf]

Table S1

Measures of Personal Resources, Social-Contextual Resources, and Mental Health and Well-being

Construct	Measure(s)	Reporter(s)[a]	Sample Item(s)[b]	Scoring	Reliability (T1/T2)
			Personal Resources		
**Self-control**	Grasmick et al. (1993)<br><br>8 items asking youth how true each statement is for them or parent about agreement that the statement describes the youth<br><br>Youth response options:<br>• Not at all true (1)<br>• A little true (2)<br>• Somewhat true (3)<br>• Mostly true (4)<br>• Always true (5)<br><br>Parent response options:<br>• Strongly disagree (1)<br>• Disagree (2)<br>• Agree (3)<br>• Strongly agree (4)	Y / P	• I often act on the spur of the moment without stopping to think. (R)<br>• Sometimes I will take a risk just for the fun of it. (R)<br>• I often do whatever brings me pleasure here and now, even at the cost of some distant goal. (R)	Average of standardized (M = 0, SD = 1) scores on youth- and parent-report measures (each scored as average across items)	Y: .65/.72<br>P: .77/.90
**Conventional values**	Belief in the Moral Order Scale from the Communities that Care Survey (Arthur et al., 2002)<br><br>4 items asking youth how true each statement is for them<br><br>Response options:<br>• Not at all true (1)<br>• A little true (2)<br>• Somewhat true (3)<br>• Mostly true (4)<br>• Completely true (5)	Y	• I think sometimes it is okay to cheat at school.<br>• I think it is important to be honest with your parents, even if they become upset or you get punished. (R)<br>• I think it is okay to take something without asking if you can get away with it.	Average across items	.58/.61
**Social skills**	Social Competencies scale of the Youth Outcome Measures Online Toolbox (adapted from Muris, 2001)<br><br>7 items asking youth how true each statement is for them<br><br>Response options:<br>• Not at all true (1)<br>• A little true (2)<br>• Somewhat true (3)<br>• Mostly true (4)<br>• Completely true (5)	Y	• I can make friends with other kids.<br>• I can stay friends with other kids.<br>• I can tell other kids what I think, even if they disagree with me.	Average across items	.70/.76

Construct	Measure(s)	Reporter(s)[a]	Sample Item(s)[b]	Scoring	Reliability (T1/T2)
***Personal Resources** (continued)*					
**Coping efficacy**	Adapted from Coping Efficacy Scale (Sandler et al., 2000)<br><br>Single item<br><br>Response options from 0 to 10 presented on a ladder:<br>• 0 (What you did, did not make things better at all) to 10 (What you did made things completely better)	Y	• Sometimes the things people do to handle their problems work really well to make the situation or how they feel better. Other times what they try doesn't work at all. Think about the difficult situations or problems you have faced in the last month. How well did what you tried for handling these situations work?	Response on the single item	NA
**Spark development**	Adapted from Benson & Scales (2009)<br><br>Single item<br><br>Response options:<br>• No, not at this time (1)<br>• Sort of (2)<br>• Yes, definitely! (3)	Y	• Some people have a special interest or hobby that they really care about. This is something that takes time and effort to learn about and do well. So it would not be just watching TV or spending time on the internet or social media (e.g., YouTube). Do you have a special interest or hobby like this?	Response on the single item	NA
**Grit**	Short Grit Scale for Children (Duckworth & Quinn, 2009)<br><br>8 items asking youth how much each statement is like them<br><br>Response options:<br>• Not like me at all (1)<br>• Not much like me (2)<br>• Somewhat like me (3)<br>• Mostly like me (4)<br>• Very much like me (5)	Y	• I am a hard worker.<br>• Setbacks (delays and obstacles) don't discourage me. I bounce back from disappointments faster than most people.<br>• New ideas and projects sometimes distract me from previous ones. (R)	Average across items	.61/.72
**Self-advocacy**	Self-Advocacy Scale (Jarjoura et al., 2023)<br><br>5 items asking youth how true each statement is for them<br><br>Response options:<br>• Not at all true (1)<br>• A little true (2)<br>• Somewhat true (3)<br>• Mostly true (4)<br>• Completely true (5)	Y	• I am good at figuring out how to get the kind of help I need to solve a problem.<br>• I can figure out how to get involved in activities that I enjoy or want to learn more about.<br>• When I want to do something new, I think of ideas for how to make it happen.	Average across items	.75/.79

Construct	Measure(s)[a]	Reporter(s)[a]	Sample Item(s)[b]	Scoring	Reliability (T1/T2)
**Hopeful future expectations**	Abbreviated version of the Hopeful Future Expectations Scale (Bowers et al., 2012)<br><br>7 items asking youth how they see each description being true for them when they are older and an adult<br><br>Response options:<br>• I'm very sure it won't be true (1)<br>• I think it probably won't be true (2)<br>• I think it probably will be true (3)<br>• I'm sure it will be true (4)	Y	• Having a job or career that you really enjoy<br>• Having enough money to buy the things you need<br>• Being healthy	Average across items	.76/.77
**Goal setting and pursuit**	Goal Orientation Scale (Child Trends, 2022)<br><br>7 items asking how much each statement describes the youth<br><br>Response options:<br>• Not at all like this child (1)<br>• A little like this child (2)<br>• Somewhat like this child (3)<br>• A lot like this child (4)<br>• Exactly like this child (5)	P	• This child has goals in his/her life.<br>• This child develops step-by-step plans to reach his/her goals.<br>• If this child sets goals, he/she takes action to reach them.	Average across items	.89/.91
Social-Contextual Resources					
**Perceived social support from family members**	Family subscale of the Multidimensional Scale of Perceived Social Support (Zimet et al., 1988)<br><br>4 items asking youth how true each statement is for them<br><br>Response options:<br>• Not at all true (1)<br>• A little true (2)<br>• Somewhat true (3)<br>• Mostly true (4)<br>• Completely true (5)	Y	• I can talk about my problems with my family.<br>• My family really tries to help me.<br>• My family is willing to help me make decisions.	Average across items	.85/.90

Construct	Measure(s)[a]	Reporter(s)[a]	Sample Item(s)[b]	Scoring	Reliability (T1/T2)
***Social-Contextual Resources** (continued)*					
**Perceived social support from friends**	Friends subscale of the Multidimensional Scale of Perceived Social Support (Zimet et al., 1988)<br><br>4 items asking youth how true each statement is for them<br><br>Response options:<br>• Not at all true (1)<br>• A little true (2)<br>• Somewhat true (3)<br>• Mostly true (4)<br>• Completely true (5)	Y	• My friends really try to help me.<br>• I can count on my friends when things go wrong.<br>• I can talk about my problems with my friends.	Average across items	.89/.93
**Perceived social support from significant other**	Significant Other subscale of the Multidimensional Scale of Perceived Social Support (Zimet et al., 1988)<br><br>4 items asking youth how true each statement is for them<br><br>Response options:<br>• Not at all true (1)<br>• A little true (2)<br>• Somewhat true (3)<br>• Mostly true (4)<br>• Completely true (5)	Y	• There is a special person who is around when I am in need.<br>• There is a special person in my life who cares about my feelings.<br>• I have a special person who is a real source of comfort to me.	Average across items	.86/.90
**Family functioning**	General Family Functioning scale of the Family Assessment Device (Epstein et al., 1983)<br><br>12 items asking parent how much they agree or disagree with each statement<br><br>Response options:<br>• Strongly disagree (1)<br>• Disagree (2)<br>• Agree (3)<br>• Strongly agree (4)	P	• Making decisions is a problem for our family. (R)<br>• Individuals are accepted for what they are.<br>• We avoid discussing our fears and concerns. (R)	Average across items	.89/.90

Construct	Measure(s)	Reporter(s)[a]	Sample Item(s)[b]	Scoring	Reliability (T1/T3)
***Social-Contextual Resources** (continued)*					
**Supportive parenting behavior**	Involvement (10 items), Positive Parenting (6 items), Poor Monitoring/Supervision (10 items), and Inconsistent Discipline (6 items) subscales of the Alabama Parenting Questionnaire (Essau et al., 2006)<br><br>Items asking how often each behavior or situation typically occurs in the youth's home<br><br>Response options:<br>• Never (1)<br>• Almost never (2)<br>• Sometimes (3)<br>• Often (4)<br>• Always (5)	P	• You have a friendly talk with this child. (Involvement)<br>• You praise this child if he/she behaves well. (Positive Parenting)<br>• This child is out with friends you don't know. (Poor Monitoring/Supervision)<br>• You threaten to punish this child and then do not actually punish him/her. (Inconsistent Discipline)	Average of standardized (*M* = 0, *SD* = 1) scores on subscales (each scored as average across items), inversion of scores for Poor Monitoring/ Supervision and Inconsistent Discipline	.68 to .83/.74 to .84
**Involvement in organized youth activities**	Herrera et al. (2007)<br><br>4 items asking whether youth has been involved in different types of activities during the past 12 months at baseline and past 18 months at follow-up<br><br>Response options:<br>• No<br>• Yes	P	• After-school programs or activities at their school (like arts, science club, music or sports)?<br>• Clubs during the school day at his/her school (like band, newspaper, drama, chorus, public speaking)?<br>• An after-school program or activity but not at his/her school (like a sports team, music lessons, Boys & Girls Club, 4H, Boy/Girl Scouts, YMCA, recreation center or a church youth group)?<br>• A leader in a school or community activity (for example serving in student council or student government)?	Number of activities with yes responses	NA
**Volunteering**	Herrera et al. (2013)<br><br>Single item asking youth if they engaged in the activity described during the past 12 months at baseline and past 18 months at follow-up<br><br>Response options:<br>• No (0)<br>• Yes (1)	Y	• Volunteered in your community	Response on the single item	NA

Construct	Measure(s)	Reporter(s)[a]	Sample Item(s)[b]	Scoring	Reliability (T1/T2)
			Mental Health and Well-being		
**Self-esteem**	Global Self-Esteem subscale of the Brief version of the Self-Esteem Questionnaire (DuBois et al., 1996)<br><br>4 items asking youth how true each statement is for them<br><br>Response options:<br>• Not at all true (1)<br>• A little true (2)<br>• Somewhat true (3)<br>• Mostly true (4)<br>• Completely true (5)	Y	• I like being just the way I am.<br>• I am happy with myself as a person.<br>• I am the kind of person I want to be.	Average across items	.83/.87
**Positive affect**	Short-form Pediatric Positive Affect Scale: Patient-Reported Outcomes Measurement Information System (PROMIS; Forrest et al., 2018)<br><br>4 items asking how often statement has been true over the past 7 days<br><br>Response options:<br>• Never (0)<br>• Almost never (1)<br>• Sometimes (2)<br>• Often (3)<br>• Almost always (4)	Y	• I felt great.<br>• I felt cheerful.<br>• I felt joyful.<br>• I felt happy.	Sum across items	.87/.88
**Life satisfaction**	Cantril (1965)<br><br>Single item asking youth how they feel about the way their life is<br><br>Response options from 0 to 10 presented on a ladder:<br>• 0 (The worst possible life) to 10 (The best possible life)	Y	• In general, where on the ladder do you feel you stand at the moment?	Response on the single item	NA

[a] Y=Youth; P=Parent. [b](R) designates an item that was reverse-scored.
