# Supplementary material for: An Investigation of Personal and Social-Contextual Resources as Mediators of the Influence of Duration of COVID-19 Pandemic Exposure and Mentoring Program Participation on the Mental Health and Well-Being of Adolescents
Source: Behav Sci (Basel). 2026 Oct 9;16(10):1857. doi: 10.3390/bs16101857 (PMC13701299; doi:10.3390/bs16101857)
Supplement: Supplementary file 1 [file behavsci-16-01857-s001.zip › Table S2.pdf]

Table S2

Baseline Measures Exhibiting Associations ($p < .10$ or Stronger) with Intervention Group Status (BBBS Program or Control) and/or Pandemic Exposure

| Measures | Associations | |
|---|---|---|
| | Intervention Group Status (1 = BBBS program; 0 = control) | Pandemic Exposure |
| Agency | --- | .247[a,b] |
| Youth Race/Ethnicity | --- | .011[a] |
| Single-adult Household | .064[c] | --- |
| Violence-related Delinquent Behavior[d] | --- | .048[e] |
| Conventional Values[f] | --- | -.047[g] |
| Hopeful Future Expectations[f] | --- | -.061[g] |
| Social Competence[f] | --- | -.050[g] |
| Self-advocacy[f] | --- | -.088 [g] |
| Grit[f] | .049 | -.048 |
| Goal-setting and Pursuit[f] | -.076[e] | --- |
| Parenting Behavior: Inconsistent Discipline[f] | --- | .076[g] |
| Involvement in Organized Youth Activities[f] | -.074[e] | -.013[g] |
| Self-esteem[f] | --- | -.095[g] |
| Positive Affect[f] | --- | -.101[g] |
| Life Satisfaction[f] | --- | -.109[g] |
| Depressive Symptoms[d] | --- | .086[g] |
| Academic Performance[d] | --- | -.128[g] |
| Career Exploration[d] | --- | -.046 [g] |
| Youth Risk Exposure[d] | --- | .095[g] |

[a]Eta-squared. [b]One agency that enrolled a relatively large number of participants and did so relatively early in the study enrollment period made an outsized contribution to this association. When excluding this agency, the association was reduced to .145 (eta-squared). [c]Phi coefficient. [d]Details regarding this measure are provided in DuBois et al. (2022). [e]Point biserial correlation. [f]Details regarding this measure are provided in Supplementary Table S1. [g]Pearson correlation.
